# Glycointeractome of Neisseria gonorrhoeae: Identification of Host Glycans Targeted by the Gonococcus To Facilitate Adherence to Cervical and Urethral Epithelial Cells

**DOI:** 10.1128/mBio.01339-19

**Published:** 2019-07-09

**Authors:** Evgeny A. Semchenko, Arun V. Everest-Dass, Freda E.-C. Jen, Tsitsi D. Mubaiwa, Christopher J. Day, Kate L. Seib

**Affiliations:** a Institute for Glycomics, Griffith University, Gold Coast, Queensland, Australia; University of Wisconsin–Madison; Nanyang Technological University

**Keywords:** *Neisseria gonorrhoeae*, adherence, carbohydrate, epithelial cells, glycobiology, gonorrhea, mannose

## Abstract

Neisseria gonorrhoeae is a significant threat to global health for which a vaccine and novel treatment options are urgently needed. Glycans expressed by human cells are commonly targeted by pathogens to facilitate interactions with the host, and thus characterization of these interactions can aid identification of bacterial receptors that can be exploited as vaccine and/or drug targets. Using glycan array analysis, we identified 247 specific interactions between N. gonorrhoeae and glycans representative of those found on human cells. Interactions included those with mannosylated, fucosylated, and sialylated glycans, glycosaminoglycans (GAGs), and glycans terminating with galactose (Gal), *N*-acetylgalactosamine (GalNAc), and *N*-acetylglucosamine (GlcNAc). By investigating the kinetics of interactions with selected glycans, we demonstrate that whole-cell N. gonorrhoeae has a high affinity for mannosylated glycans (dissociation constant [*K_D_*], 0.14 to 0.59 μM), which are expressed on the surface of cervical and urethral epithelial cells. Using chromatography coupled with mass spectrometric (MS) analysis, we identified potential mannose-binding proteins in N. gonorrhoeae. Pretreatment of cells with mannose-specific lectin (concanavalin A) or free mannose competitor (α-methyl-d-mannopyranoside) substantially reduced gonococcal adherence to epithelial cells. This suggests that N. gonorrhoeae targets mannosyl glycans to facilitate adherence to host cells and that mannosides or similar compounds have the potential to be used as a novel treatment option for N. gonorrhoeae.

## Abstract (precis)

Multidrug-resistant strains of Neisseria gonorrhoeae are emerging worldwide, and novel treatment and prevention strategies are needed. Glycans are ubiquitously expressed by all human cells and can be specifically targeted by pathogens to facilitate association with host cells. Here we identify and characterize the N. gonorrhoeae host-glycan binding profile (glycointeractome), which revealed numerous interactions, including high-affinity binding to mannosyl glycans. We identify gonococcal potential mannose-binding proteins and show that N. gonorrhoeae uses mannosyl glycans expressed on the surface of cervical and urethral epithelia to facilitate adherence. Furthermore, a mannose-binding lectin or a mannoside compound was able to reduce this adherence. By characterizing the glycointeractome of N. gonorrhoeae, we were able to elucidate a novel mechanism used by this important pathogen to interact with human cells, and this interaction could be exploited to develop novel therapeutics to treat antibiotic-resistant gonorrhea.

## INTRODUCTION

Neisseria gonorrhoeae is a highly adapted human mucosal pathogen that causes the sexually transmitted infection gonorrhea. While infections in men are typically symptomatic, infections in women are frequently asymptomatic (up to 80% of cases), which significantly increases the chance of infection not being detected and treated and thus increasing the risk of transmission and the development of sequelae (1–3). Untreated gonococcal infections can lead to pelvic inflammatory disease, neonatal complications, and infertility (4, 5) and also increase the risk of acquiring and transmitting HIV (6, 7). Treatment of N. gonorrhoeae infection is becoming problematic due to increasing antimicrobial resistance, which highlights the urgent need for the development of alternative treatment options and a vaccine (8–10). As such, interactions between host cells and the bacterium need to be characterized to aid identification of target molecules that can be exploited for antibiotic and vaccine development.

Glycans (mono- or polysaccharides) are ubiquitous in nature and can be found in all tissues and bodily fluids (e.g., blood, mucosal secretions) in humans. For example, the extracellular matrix (ECM) is rich in glycosaminoglycans (GAGs; e.g., heparan and chondroitin sulfate), proteoglycans (e.g., versican and syndecan), glycolipids (e.g., blood group antigens), and glycoproteins (e.g., mucins) (11). Glycans are involved in multiple biological processes, like cell-cell recognition, cell division, and cell signaling (12). Glycans that decorate the surface of cells are commonly referred to as the cellular glycocalyx and constitute a physical barrier between the cell and the environment (13). It is not surprising that many pathogens have evolved to interact with human glycans to facilitate association and entry into target cells and to regulate host responses (14). For example, Escherichia coli FimH initiates primary contact with human urethral cells by binding to mannosyl glycans (15). N. gonorrhoeae also binds host glycans, with several interactions described in the literature (16, 17). For instance, the gonococcus binds several human GAGs and glycoproteins, including heparin (found in serum) (18), heparan/chondroitin sulfate (found in the ECM) (19, 20), and vitronectin (found in serum and ECM) (21), which promotes association with host cells or facilitates increased resistance to serum-mediated killing. Gonococcal surface molecules that contain glycans (i.e., glycoproteins and lipooligosaccharide [LOS]) also interact with several host receptors to facilitate adherence and activation of signaling pathways (e.g., the N. gonorrhoeae LOS moiety, lacto-*N*-neotetraose, interacts with the human asialoglycoprotein receptor [hASGPR] [22], and the gonococcal pilin glycan [Gal(α1-3)-diNAcBac] interacts with human complement receptor 3 [CR3]) (17). However, the current understanding of gonococcus-host glycan-based interactions is incomplete. Here, we address this by characterizing the glycointeractome of N. gonorrhoeae by the use of glycan array analysis and surface plasmon resonance (SPR) and identify glycan-binding gonococcal proteins. To complement this study, we have screened and determined terminal glycan moieties present on the surface of human cervical and urethral epithelial cells, which were subsequently used to investigate the role of these glycans in N. gonorrhoeae-host interactions.

## RESULTS

### Glycan array analysis shows that N. gonorrhoeae binds multiple glycans from different structural and functional classes.

**FIG 1:**
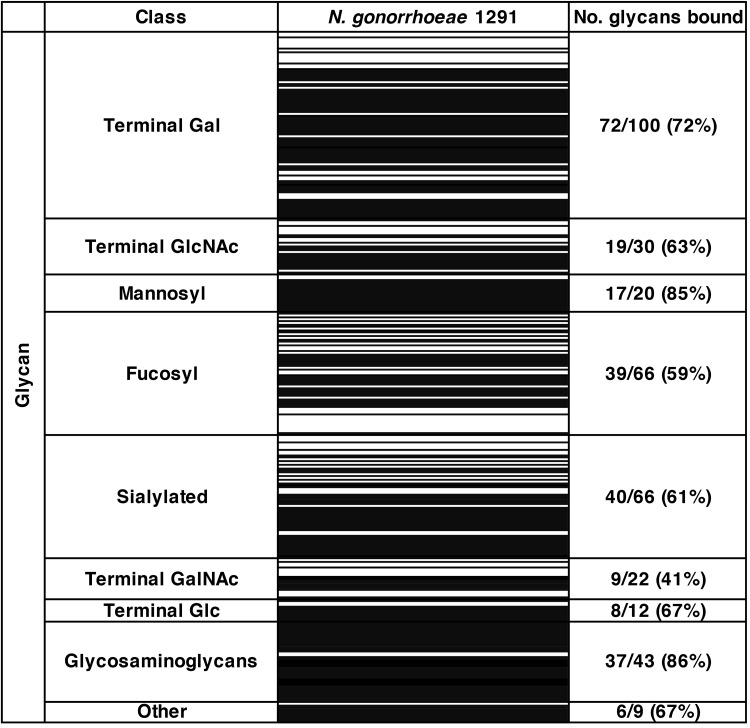
Glycan binding by Neisseria gonorrhoeae. The heat map shows binding (black bars) by whole-cell N. gonorrhoeae strain 1291 to glycans on the array (average of results from three independent experiments). Glycans are clustered into classes based on their respective terminal sugars. The number and percentage of glycans bound within each class are indicated. The full data set of glycan binding is shown in Table S1 in the supplemental material.

To ascertain the glycan binding profile of N. gonorrhoeae, glycan array analysis was performed with fluorescently labeled whole-cell bacteria and glycan arrays that display 368 structures mostly representative of glycans found on human cells (including several glycans that are similar in structure or function but differ in spacer size, chemical linkage, or chain length). N. gonorrhoeae bound to 247 glycans, including mannosylated, fucosylated, and sialylated glycans, GAGs, and GAG digests, as well as glycans terminating with glucose, galactose, *N*-acetylgalactosamine, and *N*-acetylglucosamine. In particular, a large number of GAGs and mannosylated glycans were bound, with binding to 86% and 85% of these structures on the array, respectively. Of the 20 mannosyl glycans on the array, the gonococcus bound to the 17 polysaccharides but did not bind to the three monosaccharide structures. The glycan array results for N. gonorrhoeae are summarized in Fig. 1, and a full set of results is shown in Table S1 in the supplemental material.

10.1128/mBio.01339-19.4 TABLE S1 Heat map of glycan array results for Neisseria gonorrhoeae 1291. Black indicates binding (in three independent experiments), and white indicates no binding. Binding was defined as a value greater than a 1-fold increase above mean background relative fluorescence units (RFU). The fold increase above background is indicated. HA, hyaluronic acid; ΔUA, unsaturated uronic aid. Spacers used: sp2, (CH2)3-NH-; sp3, (CH2)5-NH-; sp4, NH-(CO)CH2-NH-. Download Table S1, PDF file, 0.2 MB. Copyright © 2019 Semchenko et al. 2019 Semchenko et al. This content is distributed under the terms of the Creative Commons Attribution 4.0 International license.

### Characterization of N. gonorrhoeae-glycan interactions using SPR.

Surface plasmon resonance (SPR) analysis was used to determine the affinities of interactions between whole-cell N. gonorrhoeae and free glycans, using a subset of glycans representing the structural and functional glycan classes that were bound on the array. Whole-cell N. gonorrhoeae binds all selected glycans with a high affinity (except for Tn antigen, a negative control for which no binding was detected on the array), supporting the glycan array data. The dissociation constants (*K_D_*) calculated for these interactions were in the nanomolar to low micromolar range, as shown for terminal Gal and GlcNAc glycans, heparin, Lewis X, sialyl-Lewis X, and Lewis Y, as well as blood group antigens A, B, and O (Table 1) (SPR sensorgrams are shown in Fig. S1 in the supplemental material). The highest-affinity interaction was seen for α1-2 mannobiose, with a *K_D_* of 0.14 ± 0.06 μM.

**TABLE 1:** SPR analysis of gonococcal interactions with selected glycans^*a*^

Glycan class	Glycan index	Glycan name	Glycan structure	*K_D_* (μM)
Terminal Gal	1G	Lacto-*N*-tetraose (LNT)	Galβ1-3GlcNAcβ1-3Galβ1-4Glc	0.67 ± 0.12
	1H	Lacto-*N*-neotetraose (LNnT)	Galβ1-4GlcNAcβ1-3Galβ1-4Glc	0.51 ± 0.08
	1N	α1-3 Galactobiose	Galα1-3Gal	0.50 ± 0.12
Terminal GlcNAc	4D	Hexaacetyl chitohexaose	(GlcNAcβ1-4GlcNAc)*_n_*_ = 3_	0.61 ± 0.17
Mannosyl	5C	α1-2 Mannobiose	Manα1-2Man	0.14 ± 0.06
	5D	α1-3 Mannobiose	Manα1-3Man	0.59 ± 0.28
	5H	α1-3, α1-3, α1-6 Mannopentaose	Manα1-6(Manα1-3)Manα1-6(Manα1-3)Man	0.58 ± 0.18
Fucosyl	7K	Blood group A	GalNAcα1-3(Fucα1-2)Gal	3.55 ± 0.47
	7M	Blood group B	Galβ1-3(Fucα1-2)Gal	0.54 ± 0.06
	7F	Blood group O	(Fucα1-2)Gal	0.56 ± 0.07
	7I	Lewis X	Galβ1-4(Fucα1-3)GlcNAc	2.12 ± 0.82
	7N	Lewis Y	Fucα1-2Galβ1-4(Fucα1-3)GlcNAc	0.28 ± 0.16
Sialylated	10B	Sialyl-Lewis X	Neu5Acα2-3Galβ1-4(Fucα1-3)GlcNAc	0.22 ± 0.05
	11A	α2-3 Sialyllactose	Neu5Acα2-3Galβ1-4Glc	0.77 ± 0.1
	11B	α2-6 Sialyllactose	Neu5Acα2-6Galβ1-4Glc	0.29 ± 0.08
Terminal GalNAc	1L	Tn antigen^*b*^	GalNAcɑ-*O*-Ser	N
Glycosaminoglycans	13J	Heparin	(GlcA/IdoAα/β1-4GlcNAcɑ1-4)*_n_*_ = 200_	1.83 ± 0.8

a The affinity (dissociation constant [*K_D_*]) of the interaction between whole-cell N. gonorrhoeae and a glycan is shown.

b Tn antigen (1L) was used as a negative control as it did not bind N. gonorrhoeae on the glycan array. N, no concentration-dependent interaction detected. Sensorgrams of all interactions are shown in Fig. S1 in the supplemental material.

10.1128/mBio.01339-19.1 FIG S1 Glycan binding by N. gonorrhoeae. Representative sensorgrams from surface plasmon resonance (SPR) analysis of whole-cell N. gonorrhoeae and selected glycans. Download FIG S1, PDF file, 0.8 MB. Copyright © 2019 Semchenko et al. 2019 Semchenko et al. This content is distributed under the terms of the Creative Commons Attribution 4.0 International license.

### Identification of gonococcal mannose-binding proteins.

To identify a gonococcal mannose-binding protein(s), the bacterial cell outer membrane protein fraction was incubated with d-mannose-coupled agarose. Bound proteins were eluted and visualized using SDS-PAGE. At least 10 protein bands were detected on the Coomassie blue-stained gel, with major bands at 37 and 25 kDa (Fig. S2A). Liquid chromatography-mass spectrometry (LC-MS) analysis was performed on the total protein sample eluted from the d-mannose-coupled agarose, as well as on the 10 main bands excised from the protein gel, to enable confirmation of the most abundant mannose-binding proteins (Table 2 and Data Set S1). To exclude nonspecific protein binding to the agarose resin, a duplicate cell membrane sample containing d-mannose as a binding competitor was applied to the agarose (negative control). Minimal protein was eluted from this sample, with only three faint bands visible on the Coomassie blue-stained gel, suggesting that most of the eluted proteins interact with mannosyl glycans in a specific manner (Table 2 and Fig. S2B). Overall, using affinity chromatography (d-mannose) coupled with MS, we identified 17 proteins, which we hypothesize may be involved in gonococcal binding to mannosylated glycans.

**TABLE 2:** Gonococcal mannose-binding proteins^*a*^

Band	Accession no.	Gene^*b*^	Name	Mol wt (kDa)
1	Q5F5H1	NGO1955	Uncharacterized protein	147.2
2	Q5F6Q4	NGO1495	Transferrin-binding protein A	101.9
	Q5F845	NGO0952	TonB-dependent receptor protein	104.1
3	Q5F5W8	NGO1801	Outer membrane protein assembly factor BamA	87.9
	Q5F651	NGO1715	LPS-assembly protein LptD	87.5
	Q5F7H3	NGO1205	TonB-dependent receptor protein	84.9
4^*c*^	Q5F969	NGO0530	Putative acyl coenzyme A ligase	57.4
	Q5F4W6	NGO2178	Membrane protein insertase YidC	60.5
5	Q5F726	NGO1363	Multidrug efflux pump channel protein	50.4
6	Q5F693	NGO1669	Pilus assembly protein	45.3
7^*c*^	Q5F5V7	NGO1812	Major outer membrane protein P.IB (PorB)	37.2
	Q5F8I5	NGO0788	Genome-derived *Neisseria* antigen 1220	34.5
8	Q5F6I1	NGO1577	Outer membrane protein PIII (Rmp)	25.5
9^*c*^	Q5F9W0	NGO0277	Outer membrane protein assembly factor BamD	30.8
10	A0A0H4IV81	NGO1073a	Opacity protein	25.8
	A0A0H4IV74	NGO1040a	Opacity protein	25.6
	Q5F6N6	NGO1513	Opacity protein OpaD	26.6

a Gonococcal mannose-binding proteins from gel-excised bands as identified by liquid chromatography-mass spectrometry (LC-MS) analysis. The top protein candidates from each band are shown.

b Gene locus name from the N. gonorrhoeae strain FA1090 genome (GenBank accession no. NC_002946.2).

c These bands were also visualized on the gel of the negative-control sample but at reduced levels.

10.1128/mBio.01339-19.2 FIG S2 Gonococcal mannose-binding proteins. (A) N. gonorrhoeae 1291 mannose-binding proteins eluted from d-mannose-coupled agarose and resolved on a 4 to 12% Bis-Tris SDS-PAGE gel. (B) The negative control (cell membrane proteins with d-mannose added as a binding competitor prior to incubation with d-mannose agarose) contained negligible protein, suggesting that most of the eluted proteins interact with mannose in a specific manner. Bands (1 to 10) were excised from the gel for protein identification using mass spectrometry. Download FIG S2, PDF file, 0.8 MB. Copyright © 2019 Semchenko et al. 2019 Semchenko et al. This content is distributed under the terms of the Creative Commons Attribution 4.0 International license.

10.1128/mBio.01339-19.5 DATA SET S1 Mass spectrometry identification of gonococcal mannose-binding proteins. Liquid chromatography-mass spectrometry (LC-MS) analysis of the total protein sample (in solution) eluted from the d-mannose-coupled agarose, as well as of the 10 main bands excised from the protein gel (bands 1 to 10). Download Data Set S1, XLSX file, 0.9 MB. Copyright © 2019 Semchenko et al. 2019 Semchenko et al. This content is distributed under the terms of the Creative Commons Attribution 4.0 International license.

Three opacity proteins (NGO1073a, NGO1040a, NGO1513) were identified as the most abundant mannose-binding proteins visualized on the Coomassie blue-stained gel following elution from the mannose-coupled agarose (Table 2 and Fig. S2A, band 10), which were absent from the negative-control sample (Fig. S2B). To further investigate the role of opacity proteins in binding mannose, we analyzed the kinetics of interactions of Opa-expressing (Opa^+^) and nonexpressing (Opa^–^) N. gonorrhoeae with mannosyl glycans using SPR. We show that Opa^+^ N. gonorrhoeae binds all three mannosyl glycans tested with 6- to 27-fold-higher affinity than Opa^–^ N. gonorrhoeae (Table 3), supporting the role of opacity proteins in mannose binding.

**TABLE 3:** SPR analysis of interactions with mannosyl glycans by Opa^+^ and Opa^–^ N. gonorrhoeae

Glycan index	Glycan name	Glycan structure	*K_D_* (μM)^*a*^		Fold change
			Opa^+^	Opa^–^	
5C	α1-2 Mannobiose	Manα1-2Man	0.19 ± 0.038	5.13 ± 1.83	27
5D	α1-3 Mannobiose	Manα1-3Man	0.33 ± 0.009	3.03 ± 0.53	9
5H	α1-3, α1-3, α1-6 Mannopentaose	Manα1-6(Manα1-3)Manα1-6(Manα1-3)Man	0.25 ± 0.022	1.48 ± 0.83	6

a Opa+, Opa expressing; Opa–, Opa negative.

### Identification of glycans present on the surface of cervical and urethral epithelial cells.

Urogenital epithelial cells are a key site of gonococcal infections; however, glycans that decorate these cells are not well characterized. Therefore, we investigated the terminal glycan moieties present on the surface of transformed primary cervical epithelial cells (tCX) and urethral epithelial cells (tUEC) using flow cytometry to detect binding of fluorescently labeled lectins. Both cell types were bound by the same lectins (lectins from Colchicum autumnale [CA], Canavalia ensiformis [concanavalin A; ConA], Maackia amurensis [MAA], Robinia pseudoacacia [RPA], *Tulipa* sp. [TL], Ulex europaeus [UEA-1], and Triticum vulgaris [WGA]), suggesting that they are decorated with mannosylated, fucosylated, and sialylated structures, as well as glycans with terminal Gal, GalNAc, or GlcNAc residues. Neither tCX nor tUEC were bound by the lectin from Arachis hypogaea (PNA). These results are summarized in Table 4, and the flow cytometry histograms are shown in Fig. S3.

**TABLE 4:** tCX and tUEC surface glycans as determined by flow cytometric analysis with fluorescently labeled lectins

Lectin	Inhibiting sugar	gMFI^*a*^					
		tCX			tUEC		
		Control	Lectin	Fold change	Control	Lectin	Fold change
CA	Galβ1-4GlcNAc	1.14	3.52	3.09	1.56	4.13	2.65
ConA	Terminal αMan	1.62	95.47	58.93	2.50	260.38	104.15
MAA	NeuAcα2-3Gal	1.33	245.21	184.37	2.05	336.90	164.34
RPA	βGlc, αMan, βGlcNAc	1.62	41.65	25.71	3.21	144.82	45.12
PNA	Terminal βGal	1.14	1.41	1.24	1.56	1.46	0.94
TL	α/β-GalNAc > Gal > Fuc	1.62	69.40	42.84	3.21	578.28	180.15
UEA-1	αFuc	1.14	6.12	5.37	1.56	63.37	40.62
WGA	βGlcNAc/NeuAc	1.14	130.36	114.35	1.56	163.70	104.94

a Positive binding was determined as a ≥2-fold increase in geometric mean fluorescence intensity (gMFI) relative to that of the unlabeled cell control. Histograms of individual labeling of cells with lectins are shown in Fig. S3 in the supplemental material. tCX, cervical epithelial cells; tUEC, urethral epithelial cells.

10.1128/mBio.01339-19.3 FIG S3 Analysis of lectin binding by epithelial cells. Identification of urethral epithelial cell (tUEC) (A) and cervical epithelial cell (tCX) (B) surface glycans with fluorescein isothiocyanate (FITC)-labeled lectins using flow cytometry. A control histogram shows the overlay of the nonlabeled cell population and cells incubated with the final elution of the buffer that followed the cleanup of the FITC labeling of lectins. This demonstrates that no unreacted, residual fluorescein molecules were present in any of the lectin samples. Each panel shows a histogram from an individual experiment with a lectin (top right corner) and the glycan it recognizes (bottom label). Abbreviations: CA, Colchicum autumnale lectin; ConA, Canavalia ensiformis lectin (concanavalin A); MAA, Maackia amurensis lectin; PNA, Arachis hypogaea lectin; RPA, Robinia pseudoacacia lectin; TL, *Tulipa* sp. lectin; UEA-1, Ulex europaeus lectin; WGA, Triticum vulgaris lectin. Download FIG S3, PDF file, 0.2 MB. Copyright © 2019 Semchenko et al. 2019 Semchenko et al. This content is distributed under the terms of the Creative Commons Attribution 4.0 International license.

### N. gonorrhoeae uses host-mannosylated glycans to facilitate adherence to human epithelial cells.

**FIG 2:**
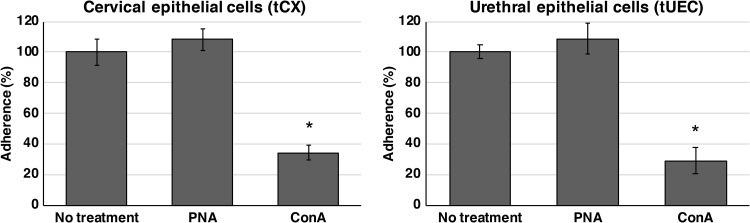
Adherence of N. gonorrhoeae to cervical epithelial cells (tCX) and urethral epithelial cells (tUEC) in the presence and absence of lectin. Gonococcal adherence is reduced in cells pretreated with the mannose-specific lectin ConA. Results are presented as the percentage of adherent bacteria from triplicate lectin-treated samples relative to that of the no-treatment control [results for no-treatment controls set at 100% are (1.7 ± 0.15) × 10^3^ and (1.4 ± 0.06) × 10^3^ adherent CFU for tCX and tUEC, respectively]. Error bars represent ±1 standard deviation. *, *P* < 0.05, calculated using a two-tailed Student's (*P* = 0.00032 and *P* = 0.0002 for tCX and tUEC, respectively). The nonbinding lectin PNA did not reduce gonococcal adherence (*P* = 0.26 and *P* = 0.24 for tCX and tUEC, respectively). Experiments were performed on at least three occasions, and representative results are shown.

N. gonorrhoeae bound several mannosyl glycans on the array (Fig. 1), and the highest-affinity interaction detected was with α1-2 mannobiose (Table 2). Mannose is expressed by various cell types relevant to mucosal pathogens, including the genital tract epithelial cells used in our adherence model (Table 4). To determine whether N. gonorrhoeae interacts with host cell mannosyl glycans for adherence, infection inhibition assays were performed with epithelial cells that were pretreated with a mannose-binding lectin (ConA) to occupy available mannosyl glycans on the surface of cells. We show that treatment of both cervical and urethral epithelial cells with ConA resulted in a 3-fold reduction in gonococcal association, with 34% and 29% adherence (relative to that of the no-treatment control), respectively (Fig. 2). No reduction in gonococcal adherence was observed in cells pretreated with the lectin PNA (Fig. 2), a negative control that does not bind cervical or urethral cells (Fig. S3).

**FIG 3:**
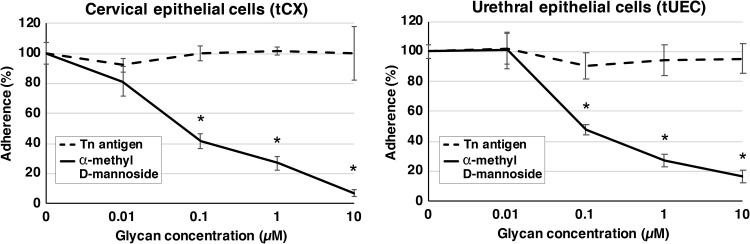
Adherence of N. gonorrhoeae to cervical epithelial cells (tCX) and urethral epithelial cells (tUEC) in the presence and absence of free glycan. Adherence of N. gonorrhoeae to tCX and tUEC is reduced in the presence of a mannose competitor, α-methyl d-mannoside. Results are presented as the percentage of adherent bacteria from triplicate glycan-treated samples relative to that of the no-treatment control [results for no-treatment controls set at 100% are (4.3 ± 0.3) × 10^3^ and (1.4 ± 0.06) × 10^3^ adherent CFU for tCX and tUEC, respectively]. Error bars represent ±1 standard deviation. *, *P* < 0.05, calculated using a two-tailed Student *t* test. Tn antigen is a negative-control nonbinding glycan that does not affect bacterial adherence (*P* > 0.19). Experiments were performed on at least three occasions, and representative results are shown.

Adherence assays were also performed with cervical and urethral cells in the presence of the free-mannose glycan, α-methyl d-mannoside, which acts as a competitor for the bacterial mannose receptor. We show that adherence to cervical cells was reduced in a concentration-dependent manner, with approximately 14-, 4-, and 2-fold reductions seen in the presence of 10, 1, and 0.1 μM α-methyl d-mannoside, respectively (Fig. 3). Similarly, adherence to urethral cells was reduced 6-, 4-, and 2-fold in the presence of 10, 1, and 0.1 μM glycan, respectively (Fig. 3). No reduction in gonococcal adherence was observed with cells pretreated with Tn antigen (Fig. 3), a negative-control glycan that was not bound by N. gonorrhoeae (Fig. 1 and Table 1). These results confirm that N. gonorrhoeae specifically targets mannosyl glycans on the surface of host cells to facilitate adherence and that these interactions can be targeted to reduce colonization and potentially inhibit gonococcal infection either alone or in combination with other compounds.

## DISCUSSION

Glycans are ubiquitous and are expressed on the surface of all human cells. They play multiple roles in cell function, cell-cell recognition, and cell signaling (12), and a large number of pathogenic organisms have evolved to exploit host glycans to facilitate adherence, to modulate cellular processes, and to avoid detection/killing by the immune system (14). Here we report the glycointeractome of N. gonorrhoeae, which reveals binding to 247 glycans representative of those found on host cells, including mannosylated, fucosylated, and sialylated glycans, GAGs, and glycans terminating with galactose (Gal), *N*-acetylgalactosamine (GalNAc), or *N*-acetylglucosamine (GlcNAc). This level of glycan interaction is similar to that seen for the closely related bacterium Neisseria meningitidis (23), which colonizes the human nasopharynx and can cause invasive diseases, including sepsis and meningitis. N. meningitidis bound to 223 glycans, from similar classes as N. gonorrhoeae, with no apparent preference shown to any kind of glycan (23). Although these organisms often colonize distinct niches in the host, they do express a number of glycan-binding protein homologues (e.g., Opc, Opa, NHBA [Neisseria Heparin Binding Antigen]) and the lipooligosaccharide (LOS), which are highly similar and likely perform a similar function in these two species (24). High levels of glycan binding has also been reported for Streptococcus pyogenes (176/343 glycans bound on the array) (25), Campylobacter jejuni (102/120 glycans bound), Salmonella enterica serovar Typhimurium (89/120 glycans bound), and Haemophilus influenzae (88/120), while Shigella flexneri (34/120 glycans bound) recognized less than half the array (26).

Surface plasmon resonance with whole-cell N. gonorrhoeae confirmed binding seen in glycan array analysis and indicated high-affinity interactions between N. gonorrhoeae and all tested glycans, including those with a terminal galactose such as lacto-*N*-neotetraose (LNnT), lacto-*N*-tetraose (LNT), and α1-3 galactobiose (*K_D_* = 0.51, 0.67, and 0.5 μM, respectively). LNT and LNnT are precursors of the ABO and P1 blood group antigens and are ubiquitously expressed on host cells (27). We also observed high-affinity interactions between the gonococcus and Lewis X, sialyl-Lewis X, and Lewis Y, as well as blood group antigens A, B, and O (*K_D_* = 0.22 to 3.55 μM). Blood group antigens are present on the surface of many human cells, including red blood cells, leukocytes, and epithelial and endothelial cells, and can be potentially targeted to facilitate association and modulation of host cellular responses (28–30). Approximately 80% of humans have blood group antigens present in their mucosal secretions (often referred to as “secretors”), allowing these glycans to be absorbed by cells for presentation on their surface (31). There may be a preference for binding human blood group antigens by N. gonorrhoeae since the gonococcus has ∼10-fold higher affinity for binding Lewis Y and sialyl-Lewis X than for binding Lewis X and ∼6-fold higher affinity for blood groups B and O than for blood group A. However, no correlation has been reported between the “secretor” status or the blood group type of a person and their predisposition to gonococcal infection (32, 33).

N. gonorrhoeae binds multiple types of GAGs, which are a diverse group of long, unbranched sulfated polysaccharides that are ubiquitous in most human tissues and secretions (e.g., serum, mucosal secretions, urine, extracellular matrix, bone, and cartilage) (34, 35). We show that the whole-cell gonococcus binds GAGs, including heparan sulfate, heparin, and their various fragment components. Interestingly, we also show that N. gonorrhoeae binds chondroitin sulfate and dermatan sulfate, which has not been reported previously. Heparan sulfate is a common GAG present mainly in the extracellular matrix and is targeted by gonococcal Opa proteins for adherence (36). Heparin is almost exclusively found in secretory granules of mast cells, which are present in all tissues but are most abundant in host-environment interfaces (e.g., cervix, urothelium, gut, and airway) (37). Mast cells play an important role in innate immunity and readily degranulate (e.g., in response to complement activation), releasing their content into the environment. Several *in vitro* studies have shown that N. gonorrhoeae binds heparin via Opa proteins, which has been linked to resistance to complement-mediated killing (18, 38). Chondroitin sulfate and dermatan sulfate are common GAGs expressed in many human tissues, including skin, cartilage, bone, endothelia, connective tissues, and extracellular matrices, which have important roles in a number of biological processes (e.g., ECM structural integrity, cell adhesion, cell signaling, neuron growth, and development) (39, 40). Different growth conditions have been shown to alter bacterial glycan binding profiles (26, 41), and future work on N. gonorrhoeae looking at different conditions may reveal glycan binding profiles relevant to specific niches colonized by the gonococcus.

N. gonorrhoeae bound 17 of 20 mannosyl glycans on the array, which differ in size, linkage, organization, spacer size, and complexity; however, all have a terminal mannose. The only mannosyl glycans not bound were monosaccharides (Manα, Manβ, and ManNAcβ), which are generally difficult to access on the array due to spatial restrictions of receptor molecules. Additional investigations of interactions between whole-cell bacteria and three mannosyl glycans (α1-2 mannobiose, α1-3 mannobiose, and α1-3, α1-3, α1-6 mannopentaose) using SPR indicated that gonococcus binds these mannosyl glycans with high affinity (*K_D_* = 0.14, 0.59, and 0.58 μM, respectively). Mannosyl glycans and in particular high-mannose glycans are abundant on the surface of human oral epithelial cells as well as in the urogenital tract (42, 43). Uroplakins, which line the surface of human urothelium (44, 45), could be important for gonococcal infection in a way similar to that of the uropathogenic E. coli, where mannosyl-glycans of uroplakin Ia are bound by FimH, which mediates bacterial adherence to urothelial cells (15, 45). Using affinity chromatography coupled with mass spectrometry, we identified several potential gonococcal mannose-binding proteins from cell membrane fractions, including several well-characterized outer membrane proteins. Of these, opacity proteins are known to bind several glycans, including heparin and heparan sulfate (36), indicating the potential for interactions with multiple glycans, which is a common feature of many glycan-binding proteins (46). Glycan binding by other identified proteins has not been reported previously and will be characterized in future studies to determine if they are directly involved in mannose binding and mannose-dependent adherence to epithelial cells or if they may have been identified due to indirect interactions with mannose (i.e., as a part of a protein complex containing mannose-binding proteins). We did confirm that opacity proteins are involved in binding mannosyl glycans, as Opa-negative gonococci were shown to have reduced binding affinity for these glycans. The mechanism of mannose binding by proteins is variable; for example, plants (47), humans (48), and E. coli FimH (49) all have different structural motifs involved in mannose binding. To identify the mechanism of mannose binding by gonococcal proteins, their structure in complex with mannose would need to be determined by nuclear magnetic resonance or X-ray crystallography.

Little is known about glycans expressed by human tissues, including human cervical and urethral epithelial cells. Therefore, to complement our studies of N. gonorrhoeae-glycan interactions, we have characterized the glycan profile of human cervical and urethral epithelial cells using fluorescently labeled lectins. Both cell types had the same lectin binding profiles (i.e., binding CA, ConA, MAA, RPA, TL, UEA-1, and WGA), indicating that they are decorated with glycans containing terminal Gal, Fuc, Man, NeuAc, GlcNAc, and GalNAc moieties, which are the same glycan types bound by N. gonorrhoeae on the glycan arrays. While similar glycans can be found on many different types of cells, their abundance and representation are unique to specific cell types and can also vary between individuals (50). For example, a study of 24 human cancer cell lines found that some lectins bind multiple cell types, while each cell line had a unique lectin binding signature (51). Interestingly, 18 of 24 cell lines are likely to express mannosyl glycans, as they were bound by a mannose-binding lectin, ConA (51). Mannosyl glycans are highly abundant in the human urogenital tract (43) and are also present on other mucosal surfaces (50, 52) that are sites of gonococcal infection. In addition to epithelial cells, human monocytes, macrophages, neutrophils, basophils, eosinophils, and dendritic and mast cells express high-mannose glycan structures (53–55). Gonococcal adherence to host epithelial cells is a multifactorial process, involving various bacterial surface structures (including pili, lipooligosaccharide, porin, and Opa proteins) and several host receptor molecules (24). Initial interactions are mediated by pili, and then pilin retraction brings the gonococci close to the cell surface, allowing Opa-mediated intimate adherence and invasion (56). Here we present the first evidence that N. gonorrhoeae targets host mannosyl glycans for adherence and that this interaction could be exploited to prevent or treat the infection. We were able to significantly reduce gonococcal adherence by pretreating cells with mannose-binding lectin (ConA) or with α-methyl d-mannoside, a commercially available mannose-binding protein antagonist (57). α-Methyl d-mannoside has been used in studies with uropathogenic E. coli, as a competimer of mannose-binding protein FimH, and was able to clear the E. coli urinary tract infection in mice (58). Currently, mannoside compounds are a subject of multiple studies and have generated substantial interest as an alternative treatment option for uropathogenic E. coli infections (15, 44, 59–61). Overall, our results suggest that mannoside compounds may also have the potential to be used as preventative/treatment options for N. gonorrhoeae.

In summary, this study describes the glycointeractome of N. gonorrhoeae, demonstrating interactions with numerous glycans that are present on host cells. We also describe interactions between the gonococcus and host mannosyl glycans, which are highly abundant on the surface of human cervical and urethral cells. We demonstrate the role of these interactions *in vitro*, being able to reduce gonococcal adherence with a mannose-specific lectin or with free mannose. This suggests that mannose-binding protein antagonists could be explored as a therapeutic to treat or potentially prevent gonococcal infection *in vivo*. We also provide evidence that the gonococcus binds various additional types of glycans, which warrants future studies to define potentially novel interactions between N. gonorrhoeae and the host cells that could be targeted for antibiotic or vaccine development.

## MATERIALS AND METHODS

### Bacterial strains and growth conditions.

Gonococcal strain 1291 (male gonococcal urethritis isolate, provided by M. A. Apicella) was grown at 37°C with 5% CO_2_ on GC agar plates (Oxoid) supplemented with 1% IsoVitalex (Becton, Dickinson) for ∼16 h. The majority of the gonococcal population investigated expressed pili and Opa, as determined by phase-contrast microscopy. For experiments with Opa-negative N. gonorrhoeae (Table 3), clear colonies were selected by phase-contrast microscopy.

### Glycan array analysis.

Glycan binding of N. gonorrhoeae was investigated using array v2.0 from the Institute for Glycomics, as described previously (23, 26). Briefly, bacteria were harvested into phosphate-buffered saline (PBS), washed twice, and fixed with 2.5% (vol/vol) formaldehyde per our optimized procedures for working with pathogenic bacteria (23). Cells were then labeled with 10 μM BODIPY 558/568 NHS ester (succinimidyl ester) (Thermo Fisher Scientific) for 15 min at 37°C and washed three times with PBS^+^ (PBS plus 1 mM CaCl_2_ and 1 mM MgCl_2_). Labeling efficiency was checked by flow cytometry. Approximately 125 μl of labeled bacteria (optical density at 600 nm [OD_600_], 0.1) was applied to the glycan array slide and hybridized for 15 min at room temperature. Following incubation, the slide was washed three time with PBS^+^, dried by centrifugation (200 × *g*, 5 min), and scanned using a ProScan array scanner (Perkin Elmer). Data were analyzed using ScanArray Express (Perkin Elmer). Positive binding was defined as binding of N. gonorrhoeae 1291 to four replicate spots per array, with each spot having a value greater than 1-fold above the average background relative fluorescence units (RFU). The background level was determined from an average of four empty spots on the array plus 3 standard deviations. Triplicate array experiments were conducted on separate occasions with new samples prepared for each experiment. Statistical analysis was performed using Student’s *t* test. Experiments with live N. gonorrhoeae showed glycan binding consistent with that seen by fixed bacteria but had higher background fluorescence (data not shown).

### Surface plasmon resonance.

The affinity and kinetics of interactions between whole-cell N. gonorrhoeae and glycans were investigated using the Biacore T200 surface plasmon resonance system (General Electric). Bacteria were grown as described above, harvested into PBS, and washed twice (2,000 × *g*, 5 min, room temperature), after which bacteria were fixed in 2.5% (vol/vol) formaldehyde for 10 min at room temperature. Bacterial cells were pelleted at 2,000 × *g* and washed three times in PBS to remove residual fixative. Fixed bacteria were made up to an OD_600_ of 0.1 (∼1 × 10^7^ CFU/ml) in 10 mM sodium acetate buffer, pH 4 (optimal pH was determined by a series of pH scout experiments), and immobilized on flow cells 2, 3, and 4 at a flow rate of 5 μl/min for total contact time of 1,200 s. Flow cell 1 (no-bacteria control) was used as a reference for nonspecific interactions between glycans and the surface of the chip. Single-cycle kinetics were used to generate the *K_D_* of interactions between whole-cell N. gonorrhoeae and free glycans. Analytes were prepared and run in degassed PBS^+^ at a flow rate of 30 μl/min for total contact times of 90 s and 600 s of dissociation. Each analyte was run twice per flow cell. All data analysis was performed using Biacore T200 Evaluation software (v3.0; GE).

### Cell culture.

E6/E7 transformed primary cervical epithelial cells (tCX) (62) and male urethral epithelial cells (tUEC) (63) were cultured in 100-mm cell culture dishes in serum-free keratinocyte medium (K-SFM; supplemented with 0.16 pg/ml epidermal growth factor [rEGF] and 25 μg/ml bovine pituitary extract [BPE]) (Gibco) and serum-free prostate cell growth medium (PrEGM) (Lonza), respectively. For infection assays, cells were seeded and grown to full confluence in cell culture-coated flat-bottom 96-well plates.

### Lectin labeling and flow cytometry.

Lectins (EY Laboratories) were labeled with NHS-fluorescein (5/6-carboxyfluorescein succinimidyl ester) per the manufacturer’s instructions (Thermo Fisher Scientific). Residual NHS molecules were quenched by adding 10 mM Tris-HCl (pH 7) to the labeling reaction. The reaction buffer was exchanged for HBSS^+^ (Hanks buffered salt solution plus 1 mM CaCl_2_ and 1 mM MgCl_2_) five times using 10-kDa Amicon Ultra 0.5-ml centrifugal filters (Merck). Protein concentration and labeling efficiency were determined using a NanoDrop spectrophotometer (Thermo Fisher Scientific). tCX and tUEC monolayers were dissociated with TrypLE (Gibco) and harvested into HBSS. Cells were washed twice in HBSS^+^ containing 0.1% (vol/vol) bovine serum albumin (BSA). Approximately 500,000 cells were resuspended in 1 ml of HBSS^+^ containing 100 μg/ml of labeled lectin. Cells were incubated for 15 min at 37°C and washed three times in HBSS^+^. Lectin-bound tCX and tUEC were detected using a CyAn ADP flow cytometer (Beckman Coulter). Data analysis was performed using FlowJo (Tree Star). Unlabeled cells, or cells treated with the buffer from the final elution step, were used as negative controls.

### Cell infection assays.

Infection assays with tCX and tUEC were carried out as described previously (64), with the following modifications. N. gonorrhoeae 1291 inoculum was prepared by harvesting bacteria into HBSS, washing three times, and bacteria adjusted to an OD_600_ of 0.1 (∼1 × 10^7^ CFU/ml) in K-SFM or PrEGM. For adherence blocking with lectins, cell monolayers were washed once with HBSS^+^ and treated with 10 μg of lectins (in the respective cell medium) for 15 min at 37°C, after which cells were washed three times with HBSS. To each well, 45 μl of cell medium was added and the plate was briefly incubated at 37°C prior to subsequent infection with N. gonorrhoeae. For adherence blocking with glycans, cell monolayers were prepared as described above, 45 μl of cell medium containing the required concentration of glycan (0.01 to 10 μM α-methyl d-mannoside or Tn antigen) was added, and the plate was incubated for 5 min at 37°C, prior to infection with N. gonorrhoeae. All infection assays were initiated by adding 5 μl of bacterial inoculum to each well and incubating the plate for 10 min at 37°C. The contents of the wells were removed, and cell monolayers were washed three times with HBSS. To each well, 25 μl of TrypLE was added and the plate was incubated for 2 min at 37°C to facilitate the dissociation of cells. Subsequently, 25 μl of 1% (vol/vol) saponin in HBSS was added to each well and the contents were harvested by vigorous pipetting. The cell lysates were serially diluted and plated onto GC agar, and the total number of CFU per well was calculated. Results were calculated as the average number of CFU from three replicate wells, and data are presented as the percentage of adherent bacteria relative to that of no-treatment control wells (i.e., no lectin or glycan added). Each experiment was performed three times, and statistical analysis was performed using analysis of variance (ANOVA) and Student's *t* test.

### Isolation of N. gonorrhoeae mannose-binding proteins and sample preparation for MS.

Membranes were isolated by solubilizing the N. gonorrhoeae lysed cell pellet in 137 mM NaCl, 20 mM Tris-HCl, 1 mM CaCl_2_/MgCl_2_, and 1% (vol/vol) Triton X-100. Two soluble fraction samples (the test sample and the negative-control sample treated with 0.2 M d-mannose) were prepared and applied to d-mannose agarose (Sigma-Aldrich) (4°C, 16 h). Agarose was thoroughly washed, and then bound proteins were eluted in 2 M NaCl and resolved on a 4 to 12% Bis-Tris polyacrylamide gel (Thermo). For in-gel trypsinization of Coomassie blue-stained protein bands, excised protein bands were reduced with 10 mM dithiothreitol (DTT), alkylated with 50 mM iodoacetamide, and digested with trypsin (100 ng) for 16 h at 37°C. Peptides were extracted and reconstituted in 0.1% formic acid. Additionally, in-solution digests were set up with the eluted membrane proteins. In-solution samples were digested with trypsin (1 μg) for 16 h at 37°C. The digested peptides were desalted using C_18_ Ziptips (Millipore), according to the manufacturer’s instructions.

### Nano-LC-MS/MS analysis.

Peptide samples were analyzed by reverse-phase high-performance liquid chromatography–electrospray ionization–tandem mass spectrometry (HPLC-ESI-MS/MS) using a Dionex UltiMate 3000 RSLC nano-LC system (Thermo Scientific) connected to an Orbitrap Fusion Tribrid mass spectrometer (Thermo Scientific). The mobile phase consisted of 0.1% formic acid (A) and 0.1% formic acid in 80% acetonitrile (B). The autosampler was operated in microliter-pickup injection mode, filling a 20-μl loop with 3 μl of analyte per injection. The sample was loaded onto a reversed-phase trap (Acclaim PepMap 100, 100-μm inside diameter [i.d.] by 2 cm, 5-μm C_18_ particles) with 0.1% trifluoroacetic acid (TFA) at a flow rate of 6 μl/min. A series of nanoflow gradients (flow rate, 300 nl/min) was used to back-flush the trapped samples onto the nano-LC column (Acclaim PepMap RSLC; 75 μm i.d. by 15 cm, 2-μm C_18_ particles) for separation. The peptides were eluted using the following gradient: 2% solvent B in solvent A (from 0 to 6 min), 2% to 10% solvent B in solvent A (from 6 to 13 min), 10% to 30% solvent B in solvent A (from 13 to 63 min), 30% to 50% solvent B in solvent A (from 63 to 70 min), 50% to 95% solvent B in solvent A (from 70 to 72 min), and 95% solvent B in solvent A (from 72 to 78 min). The analysis was performed with a total run time of 90 min, including mobile-phase equilibration. Data acquisition was performed in the data-dependent mode (DDA), MS1 scans (*m/z* 400 to 1,700) were performed at a resolution of 60,000 with a 1 × 10^6^ AGC (automatic gain control) target and a maximum injection time of 75 ms. Peptide precursors with a charge state of 2 to 7 were sampled for MS2. The instrument was run in top speed mode with a cycle time of 3 s. Dynamic exclusion was enabled with the following settings: repeat count, 1; exclusion duration, 60 s; mass tolerance, ±10 ppm. MS/MS was performed by isolation at 2.0 Th with quadrupole isolation. Higher-energy collision dissociation (HCD) fragmentation was carried out at 30% normalized collision energy and 10% stepped collision energy. The tandem mass spectra were analyzed with a resolution of 30,000, the MS2 AGC target was set to 5 × 10^4^, and the maximum injection time was 250 ms.

### Database search and analyses.

Proteome Discoverer (PD, version 2.2; Thermo Scientific) was used to perform searches against the Neisseria gonorrhoeae FA 1090 protein database (UniProt Proteome ID UP000000535, 2,106 entries, released 24 May 2018). The SEQUEST-HT program was implemented in Proteome Discovery for all MS raw files. The search parameters used were as follows: 10 ppm tolerance for precursor ion masses, 0.02 Da tolerance for fragment ion masses. Two missed cleavages were allowed for fully tryptic peptides. Carbamidomethylation of cysteines (+57 Da) was set as a fixed modification, and methionine oxidation (+16 Da) was set as a variable modification. Percolator was used to score and rank spectral matches using a 1% false discovery rate. Protein hits were selected using the following criteria: molecular weight (approximate to one observed on an SDS-PAGE gel), protein localization (membrane), and top five matches with highest posterior error probability (PEP) score.

## References

[1] Edwards JL, Butler EK 2011 The pathobiology of *Neisseria gonorrhoeae* lower female genital tract infection. Front Microbiol 2:102. doi:10.3389/fmicb.2011.00102. 21747805 PMC3129011

[2] Edwards JL, Jennings MP, Apicella MA, Seib KL 2016 Is gonococcal disease preventable? The importance of understanding immunity and pathogenesis in vaccine development. Crit Rev Microbiol 42:928–941. doi:10.3109/1040841X.2015. 26805040 PMC4958600

[3] Semchenko EA, Seib KL 2016 Intractable problems require novel solutions: it's time to get serious about developing a gonorrhoea vaccine. Sex Transm Infect 92:561–562. doi:10.1136/sextrans-2015-052378. 27037185 PMC5256376

[4] Westrom L, Joesoef R, Reynolds G, Hagdu A, Thompson SE 1992 Pelvic inflammatory disease and fertility. A cohort study of 1,844 women with laparoscopically verified disease and 657 control women with normal laparoscopic results. Sex Transm Dis 19:185–192. doi:10.1097/00007435-199207000-00001. 1411832

[5] Woods CR 2005 Gonococcal infections in neonates and young children. Semin Pediatr Infect Dis 16:258–270. doi:10.1053/j.spid.2005.06.006. 16210106

[6] Galvin SR, Cohen MS 2004 The role of sexually transmitted diseases in HIV transmission. Nat Rev Microbiol 2:33–42. doi:10.1038/nrmicro794. 15035007

[7] Xu SX, Leontyev D, Kaul R, Gray-Owen SD 2018 *Neisseria gonorrhoeae* co-infection exacerbates vaginal HIV shedding without affecting systemic viral loads in human CD34+ engrafted mice. PLoS One 13:e0191672. doi:10.1371/journal.pone.0191672. 29360873 PMC5779692

[8] Unemo M, Shafer WM 2011 Antibiotic resistance in *Neisseria gonorrhoeae*: origin, evolution, and lessons learned for the future. Ann N Y Acad Sci 1230:E19–E28. doi:10.1111/j.1749-6632.2011.06215.x. 22239555 PMC4510988

[9] CDC. 16 9 2013 Antibiotic resistance threats in the United States, 2013. http://www.cdc.gov/drugresistance/threat-report-2013/pdf/ar-threats-2013-508.pdf. Accessed 7 March.

[10] WHO. 2011 Prevalence and incidence of selected sexually transmitted infections. *Chlamydia trachomatis, Neisseria gonorrhoeae*, syphilis and *Trichomonas vaginalis*. Methods and results used by WHO to generate 2005 estimates. http://www.who.int/reproductivehealth/publications/rtis/9789241502450/en/. Accessed 7 March.

[11] Rudd P, Karlsson NG, Khoo KH, Packer NH 2015 Glycomics and glycoproteomics, p 653–666. *In* Varki A, Cummings RD, Esko JD, Stanley P, Hart GW, Aebi M, Darvill AG, Kinoshita T, Packer NH, Prestegard JH, Schnaar RL, Seeberger PH (ed), Essentials of glycobiology. Cold Spring Harbor, New York, NY. doi:10.1101/glycobiology.3e.051.

[12] Varki A 2017 Biological roles of glycans. Glycobiology 27:3–49. doi:10.1093/glycob/cww086. 27558841 PMC5884436

[13] Springer SA, Gagneux P 2016 Glycomics: revealing the dynamic ecology and evolution of sugar molecules. J Proteomics 135:90–100. doi:10.1016/j.jprot.2015.11.022. 26626628 PMC4762723

[14] Poole J, Day CJ, von Itzstein M, Paton JC, Jennings MP 2018 Glycointeractions in bacterial pathogenesis. Nat Rev Microbiol 16:440–452. doi:10.1038/s41579-018-0007-2. 29674747

[15] Krogfelt KA, Bergmans H, Klemm P 1990 Direct evidence that the FimH protein is the mannose-specific adhesin of *Escherichia coli* type 1 fimbriae. Infect Immun 58:1995–1998. 1971261 10.1128/iai.58.6.1995-1998.1990 PMC258756

[16] Mubaiwa TD, Semchenko EA, Hartley-Tassell LE, Day CJ, Jennings MP, Seib KL 2017 The sweet side of the pathogenic Neisseria: the role of glycan interactions in colonisation and disease. Pathog Dis 75:ftx063. doi:10.1093/femspd/ftx063. PMC5808653 28633281

[17] Jennings MP, Jen FE, Roddam LF, Apicella MA, Edwards JL 2011 *Neisseria gonorrhoeae* pilin glycan contributes to CR3 activation during challenge of primary cervical epithelial cells. Cell Microbiol 13:885–896. doi:10.1111/j.1462-5822.2011.01586.x. 21371235 PMC3889163

[18] Chen T, Swanson J, Wilson J, Belland RJ 1995 Heparin protects Opa^+^ *Neisseria gonorrhoeae* from the bactericidal action of normal human serum. Infect Immun 63:1790–1795. 7729887 10.1128/iai.63.5.1790-1795.1995 PMC173225

[19] van Putten JP, Paul SM 1995 Binding of syndecan-like cell surface proteoglycan receptors is required for *Neisseria gonorrhoeae* entry into human mucosal cells. EMBO J 14:2144–2154. doi:10.1002/j.1460-2075.1995.tb07208.x. 7774572 PMC398320

[20] Grant CC, Bos MP, Belland RJ 1999 Proteoglycan receptor binding by *Neisseria gonorrhoeae* MS11 is determined by the HV-1 region of OpaA. Mol Microbiol 32:233–242. doi:10.1046/j.1365-2958.1999.01293.x. 10231481

[21] Gomez-Duarte OG, Dehio M, Guzman CA, Chhatwal GS, Dehio C, Meyer TF 1997 Binding of vitronectin to opa-expressing *Neisseria gonorrhoeae* mediates invasion of HeLa cells. Infect Immun 65:3857–3866. 9284164 10.1128/iai.65.9.3857-3866.1997 PMC175551

[22] Harvey HA, Porat N, Campbell CA, Jennings M, Gibson BW, Phillips NJ, Apicella MA, Blake MS 2000 Gonococcal lipooligosaccharide is a ligand for the asialoglycoprotein receptor on human sperm. Mol Microbiol 36:1059–1070. doi:10.1046/j.1365-2958.2000.01938.x. 10844691

[23] Mubaiwa TD, Hartley-Tassell LE, Semchenko EA, Jen FE, Srikhanta YN, Day CJ, Jennings MP, Seib KL 2017 The glycointeractome of serogroup B *Neisseria meningitidis* strain MC58. Sci Rep 7:5693. doi:10.1038/s41598-017-05894-w. 28720847 PMC5515891

[24] Hung MC, Christodoulides M 2013 The biology of Neisseria adhesins. Biology (Basel) 2:1054–1109. doi:10.3390/biology2031054. 24833056 PMC3960869

[25] De Oliveira DM, Hartley-Tassell L, Everest-Dass A, Day CJ, Dabbs RA, Ve T, Kobe B, Nizet V, Packer NH, Walker MJ, Jennings MP, Sanderson-Smith ML 2017 Blood group antigen recognition via the group A streptococcal M protein mediates host colonization. mBio 8:e02237-16. 28119471 10.1128/mBio.02237-16 PMC5263248

[26] Day CJ, Tran EN, Semchenko EA, Tram G, Hartley-Tassell LE, Ng PS, King RM, Ulanovsky R, McAtamney S, Apicella MA, Tiralongo J, Morona R, Korolik V, Jennings MP 2015 Glycan:glycan interactions: high affinity biomolecular interactions that can mediate binding of pathogenic bacteria to host cells. Proc Natl Acad Sci U S A 112:E7266–E7275. doi:10.1073/pnas.1421082112. 26676578 PMC4702957

[27] Nishihara S, Hiraga T, Ikehara Y, Kudo T, Iwasaki H, Morozumi K, Akamatsu S, Tachikawa T, Hisashi N 1999 Molecular mechanisms of expression of Lewis b antigen and other type I Lewis antigens in human colorectal cancer. Glycobiology 9:607–616. doi:10.1093/glycob/9.6.607. 10336994

[28] Holborow EJ, Brown PC, Glynn LE, Hawes MD, Gresham GA, O'Brien TF, Coombs RR 1960 The distribution of the blood group A antigen in human tissues. Br J Exp Pathol 41:430–437. 14402554 PMC2083207

[29] Blackwell CC 1989 The role of ABO blood groups and secretor status in host defences. FEMS Microbiol Immunol 1:341–349. doi:10.1111/j.1574-6968.1989.tb02419.x. 2698729

[30] Dunstan RA 1986 Status of major red cell blood group antigens on neutrophils, lymphocytes and monocytes. Br J Haematol 62:301–309. doi:10.1111/j.1365-2141.1986.tb02933.x. 3511947

[31] Metgud R, Khajuria N, Mamta, Ramesh G 2016 Evaluation of the secretor status of ABO blood group antigens in saliva among Southern Rajasthan population using absorption inhibition method. J Clin Diagn Res 10:ZC01–3. doi:10.7860/JCDR/2016/11598.7161. PMC4800640 27042574

[32] Chanzu NM, Mwanda W, Oyugi J, Anzala O 2015 Mucosal blood group antigen expression profiles and HIV infections: a study among female sex workers in Kenya. PLoS One 10:e0133049. doi:10.1371/journal.pone.0133049. 26186209 PMC4505875

[33] Perry HE, Franklin RA, Bray SJ, Lo MK, Svensson LA, Henry SM 2007 A novel study of association between Neisseria gonorrhoeae and the human carbohydrate blood groups. Immunohematology 23:100–104. 18284299

[34] Staprans I, Felts JM 1985 Isolation and characterization of glycosaminoglycans in human plasma. J Clin Invest 76:1984–1991. doi:10.1172/JCI112198. 4056061 PMC424260

[35] Sarrazin S, Lamanna WC, Esko JD 2011 Heparan sulfate proteoglycans. Cold Spring Harb Perspect Biol 3:a004952. doi:10.1101/cshperspect.a004952. 21690215 PMC3119907

[36] Chen T, Belland RJ, Wilson J, Swanson J 1995 Adherence of pilus^–^ Opa^+^ gonococci to epithelial cells in vitro involves heparan sulfate. J Exp Med 182:511–517. doi:10.1084/jem.182.2.511. 7629509 PMC2192128

[37] Wernersson S, Pejler G 2014 Mast cell secretory granules: armed for battle. Nat Rev Immunol 14:478–494. doi:10.1038/nri3690. 24903914

[38] Duensing TD, Putten JP 1998 Vitronectin binds to the gonococcal adhesin OpaA through a glycosaminoglycan molecular bridge. Biochem J 334:133–139. doi:10.1042/bj3340133. 9693112 PMC1219671

[39] Trowbridge JM, Gallo RL 2002 Dermatan sulfate: new functions from an old glycosaminoglycan. Glycobiology 12:117R–125R. doi:10.1093/glycob/cwf066. 12213784

[40] Yamada S, Sugahara K 2008 Potential therapeutic application of chondroitin sulfate/dermatan sulfate. Curr Drug Discov Technol 5:289–301. doi:10.2174/157016308786733564. 19075609

[41] Linden S, Mahdavi J, Hedenbro J, Boren T, Carlstedt I 2004 Effects of pH on Helicobacter pylori binding to human gastric mucins: identification of binding to non-MUC5AC mucins. Biochem J 384:263–270. doi:10.1042/BJ20040402. 15260802 PMC1134109

[42] Everest-Dass AV, Jin D, Thaysen-Andersen M, Nevalainen H, Kolarich D, Packer NH 2012 Comparative structural analysis of the glycosylation of salivary and buccal cell proteins: innate protection against infection by *Candida albicans*. Glycobiology 22:1465–1479. doi:10.1093/glycob/cws112. 22833316

[43] Kątnik-Prastowska I, Lis J, Matejuk A 2014 Glycosylation of uroplakins. Implications for bladder physiopathology. Glycoconj J 31:623–636. doi:10.1007/s10719-014-9564-4. 25394961 PMC4245495

[44] Zhou G, Mo WJ, Sebbel P, Min G, Neubert TA, Glockshuber R, Wu XR, Sun TT, Kong XP 2001 Uroplakin Ia is the urothelial receptor for uropathogenic *Escherichia coli:* evidence from in vitro FimH binding. J Cell Sci 114:4095–4103. 11739641 10.1242/jcs.114.22.4095

[45] Xie B, Zhou G, Chan SY, Shapiro E, Kong XP, Wu XR, Sun TT, Costello CE 2006 Distinct glycan structures of uroplakins Ia and Ib: structural basis for the selective binding of FimH adhesin to uroplakin Ia. J Biol Chem 281:14644–14653. doi:10.1074/jbc.M600877200. 16567801

[46] Mubaiwa TD, Hartley-Tassell LE, Semchenko EA, Day CJ, Jennings MP, Seib KL 2018 The Bexsero *Neisseria meningitidis* serogroup B vaccine antigen NHBA is a high-affinity chondroitin sulfate binding protein. Sci Rep 8:6512. doi:10.1038/s41598-018-24639-x. 29695781 PMC5916922

[47] Liu W, Yang N, Ding J, Huang RH, Hu Z, Wang DC 2005 Structural mechanism governing the quaternary organization of monocot mannose-binding lectin revealed by the novel monomeric structure of an orchid lectin. J Biol Chem 280:14865–14876. doi:10.1074/jbc.M411634200. 15649901

[48] Sheriff S, Chang CY, Ezekowitz RA 1994 Human mannose-binding protein carbohydrate recognition domain trimerizes through a triple alpha-helical coiled-coil. Nat Struct Biol 1:789–794. doi:10.1038/nsb1194-789. 7634089

[49] Bouckaert J, Berglund J, Schembri M, De Genst E, Cools L, Wuhrer M, Hung CS, Pinkner J, Slattegard R, Zavialov A, Choudhury D, Langermann S, Hultgren SJ, Wyns L, Klemm P, Oscarson S, Knight SD, De Greve H 2005 Receptor binding studies disclose a novel class of high-affinity inhibitors of the Escherichia coli FimH adhesin. Mol Microbiol 55:441–455. doi:10.1111/j.1365-2958.2004.04415.x. 15659162

[50] Everest-Dass AV, Kolarich D, Pascovici D, Packer NH 2017 Blood group antigen expression is involved in *C. albicans* interaction with buccal epithelial cells. Glycoconj J 34:31–50. doi:10.1007/s10719-016-9726-7. 27639389

[51] Tao SC, Li Y, Zhou J, Qian J, Schnaar RL, Zhang Y, Goldstein IJ, Zhu H, Schneck JP 2008 Lectin microarrays identify cell-specific and functionally significant cell surface glycan markers. Glycobiology 18:761–769. doi:10.1093/glycob/cwn063. 18625848 PMC2733773

[52] Woodward AM, Lehoux S, Mantelli F, Di Zazzo A, Brockhausen I, Bonini S, Argüeso P 2019 Inflammatory stress causes N-glycan processing deficiency in ocular autoimmune disease. Am J Pathol 189:283–294. doi:10.1016/j.ajpath.2018.10.012. 30448401 PMC6360353

[53] Bax M, Garcia-Vallejo JJ, Jang-Lee J, North SJ, Gilmartin TJ, Hernandez G, Crocker PR, Leffler H, Head SR, Haslam SM, Dell A, van Kooyk Y 2007 Dendritic cell maturation results in pronounced changes in glycan expression affecting recognition by siglecs and galectins. J Immunol 179:8216–8224. doi:10.4049/jimmunol.179.12.8216. 18056365

[54] North SJ, von Gunten S, Antonopoulos A, Trollope A, MacGlashan DW Jr, Jang-Lee J, Dell A, Metcalfe DD, Kirshenbaum AS, Bochner BS, Haslam SM 2012 Glycomic analysis of human mast cells, eosinophils and basophils. Glycobiology 22:12–22. doi:10.1093/glycob/cwr089. 21725073 PMC3230278

[55] Babu P, North SJ, Jang-Lee J, Chalabi S, Mackerness K, Stowell SR, Cummings RD, Rankin S, Dell A, Haslam SM 2009 Structural characterisation of neutrophil glycans by ultra sensitive mass spectrometric glycomics methodology. Glycoconj J 26:975–986. doi:10.1007/s10719-008-9146-4. 18587645 PMC2791480

[56] Quillin SJ, Seifert HS 2018 *Neisseria gonorrhoeae* host adaptation and pathogenesis. Nat Rev Microbiol 16:226–240. doi:10.1038/nrmicro.2017.169. 29430011 PMC6329377

[57] Welch KT, Turner TA, Preast CE 2008 Rational design of novel glycomimetics: inhibitors of concanavalin A. Bioorg Med Chem Lett 18:6573–6575. doi:10.1016/j.bmcl.2008.09.095. 18990567

[58] Aronson M, Medalia O, Schori L, Mirelman D, Sharon N, Ofek I 1979 Prevention of colonization of the urinary tract of mice with Escherichia coli by blocking of bacterial adherence with methyl alpha-d-mannopyranoside. J Infect Dis 139:329–332. doi:10.1093/infdis/139.3.329. 376757

[59] Han Z, Pinkner JS, Ford B, Obermann R, Nolan W, Wildman SA, Hobbs D, Ellenberger T, Cusumano CK, Hultgren SJ, Janetka JW 2010 Structure-based drug design and optimization of mannoside bacterial FimH antagonists. J Med Chem 53:4779–4792. doi:10.1021/jm100438s. 20507142 PMC2894565

[60] Spaulding CN, Klein RD, Ruer S, Kau AL, Schreiber HL, Cusumano ZT, Dodson KW, Pinkner JS, Fremont DH, Janetka JW, Remaut H, Gordon JI, Hultgren SJ 2017 Selective depletion of uropathogenic E. coli from the gut by a FimH antagonist. Nature 546:528–532. doi:10.1038/nature22972. 28614296 PMC5654549

[61] Cusumano CK, Pinkner JS, Han Z, Greene SE, Ford BA, Crowley JR, Henderson JP, Janetka JW, Hultgren SJ 2011 Treatment and prevention of urinary tract infection with orally active FimH inhibitors. Sci Transl Med 3:109ra115. doi:10.1126/scitranslmed.3003021. PMC3694776 22089451

[62] Potter AJ, Kidd SP, Edwards JL, Falsetta ML, Apicella MA, Jennings MP, McEwan AG 2009 Thioredoxin reductase is essential for protection of Neisseria gonorrhoeae against killing by nitric oxide and for bacterial growth during interaction with cervical epithelial cells. J Infect Dis 199:227–235. doi:10.1086/595737. 19032106 PMC2748770

[63] Harvey HA, Post DM, Apicella MA 2002 Immortalization of human urethral epithelial cells: a model for the study of the pathogenesis of and the inflammatory cytokine response to Neisseria gonorrhoeae infection. Infect Immun 70:5808–5815. doi:10.1128/IAI.70.10.5808-5815.2002. 12228311 PMC128333

[64] Semchenko EA, Day CJ, Seib KL 2017 MetQ of Neisseria gonorrhoeae is a surface-expressed antigen that elicits bactericidal and functional blocking antibodies. Infect Immun 85:e00898-16. doi:10.1128/IAI.00898-16. 27895130 PMC5278169

