# Supplementary material for: Glycointeractome of Neisseria gonorrhoeae: Identification of Host Glycans Targeted by the Gonococcus To Facilitate Adherence to Cervical and Urethral Epithelial Cells
Source: mBio. 2019 Jul 9;10(4):e01339-19. doi: 10.1128/mBio.01339-19 (PMC6747729; doi:10.1128/mBio.01339-19)
Supplement: TABLE S1 [file mBio.01339-19-st001.pdf]

The glycointeractome of *Neisseria gonorrhoeae* – identification of host glycans targeted by the gonococcus to facilitate adherence to cervical and urethral epithelial cells

Evgeny A. Semchenko, Arun V. Everest-Dass, Freda E.-C. Jen, Tsitsi D. Mubaiwa, Christopher J. Day and Kate L. Seib

Table S1. **Heat map of glycan array results for *Neisseria gonorrhoeae* 1291.**
Black indicates binding (in three independent experiments) and white indicates no binding. Binding was defined as a value greater than 1-fold increase above mean background relative fluorescence units (RFU). Fold-increase above background is indicated. HA - hyaluronic acid; ΔUA - unsaturated uronic aid. Spacers used: sp2, (CH2)3-NH- ; sp3, (CH2)5-NH- ; sp4, NH-(CO)CH2-NH-.

**Class**	**Index**	**Structure**	***N. gonorrhoeae* 1291**
Terminal galactose	2	Galα-sp3	
	3	Galβ-sp3	
	37	3-O-Su-Galβ-sp3	
	75	Galα1-2Galβ-sp3	1.16
	76	Galα1-3Galβ-sp3	
	77	Galα1-3GalNAcβ-sp3	
	78	Galα1-3GalNAcα-sp3	
	80	Galα1-3GlcNAcβ-sp3	
	81	Galα1-4GlcNAcβ-sp3	
	83	Galα1-6Glcβ-sp4	1.00
	84	Galβ1-2Galβ-sp3	
	85	Galβ1-3GlcNaAcβ-sp3	1.79
	87	Galβ1-3Galβ-sp3	
	88	Galβ1-3GalNAcβ-sp3	
	89	Galβ1-3GalNAcα-sp3	
	93	Galβ1-4Glcβ-sp4	
	94	Galβ1-4Galβ-sp4	
	97	Galβ1-4GlcNAcβ-sp3	1.46
	100	Galβ1-6Galβ-sp4	
	145	Galβ1-3(6-O-Su)GlcNAcβ-sp3	
	146	Galβ1-4(6-O-Su)Glcβ-sp2	1.68
	147	Galβ1-4(6-O-Su)GlcNAcβ-sp3	1.36
	150	3-O-Su-Galβ1-3GalNAcα-sp3	5.23
	151	6-O-Su-Galβ1-3GalNAcα-sp3	2.26
	152	3-O-Su-Galβ1-4Glcβ-sp2	4.05
	153	6-O-Su-Galβ1-4Glcβ-sp2	1.70
	155	3-O-Su-Galβ1-3GlcNAcβ-sp3	2.46

	157	3-O-Su-Galβ1-4GlcNAcβ-sp3	
	159	4-O-Su-Galβ1-4GlcNAcβ-sp3	1.41
	161	6-O-Su-Galβ1-3GlcNAcβ-sp3	1.91
	163	6-O-Su-Galβ1-4GlcNAcβ-sp3	
	176	3-O-Su-Galβ1-4(6-O-Su)Glcβ-sp2	5.69
	177	3-O-Su-Galβ1-4(6-O-Su)GlcNAcβ-sp2	2.71
	178	6-O-Su-Galβ1-4(6-O-Su)Glcβ-sp2	2.44
	179	6-O-Su-Galβ1-3(6-O-Su)GlcNAcβ-sp2	2.92
	180	6-O-Su-Galβ1-4(6-O-Su)GlcNAcβ-sp2	2.30
	181	3,4-O-Su$_2$-Galβ1-4GlcNAcβ-sp3	1.46
	182	3,6-O-Su$_2$-Galβ1-4GlcNAcβ-sp2	4.42
	183	4,6-O-Su$_2$-Galβ1-4GlcNAcβ-sp2	3.42
	184	4,6-O-Su$_2$-Galβ1-4GlcNAcβ-sp3	2.70
	189	3,6-O-Su$_2$-Galβ1-4(6-O-Su)GlcNAcβ-sp2	1.45
	201	3,4-O-Su$_2$-Galβ1-4GlcNAcβ-sp3	2.66
	203	Galβ1-4(6-O-Su)GlcNAcβ-sp2	2.58
	220	Galα1-3Galβ1-4Glcβ-sp2	5.33
	222	Galα1-3Galβ1-4GlcNAcβ-sp3	
	224	Galα1-4Galβ1-4Glcβ-sp3	3.84
	225	Galα1-4Galβ1-4GlcNAc-sp2	1.44
	228	Galβ1-2Galα1-4GlcNAcβ-sp4	1.81
	229	Galβ1-3Galβ1-4GlcNAcβ-sp4	2.06
	231	Galβ1-4GlcNAcβ1-3GalNAcα-sp3	1.10
	232	Galβ1-4GlcNAcβ1-6GalNAcα-sp3	1.68
	254	Galβ1-3(GlcNAcβ1-6)GalNAcα-sp3	1.67
	262	Galβ1-3GalNAcβ1-3Gal-sp4	1.26
	264	Galβ1-4Galβ1-4GlcNAc-sp3	1.17
	373	Galα1-3Galβ1-4GlcNAcβ1-3Galβ-sp3	1.43
	375	Galα1-4GlcNAcβ1-3Galβ1-4GlcNAcβ-sp3	1.47
	376	Galβ1-3GlcNAcβ1-3Galβ1-4Glcβ-sp4	
	377	Galβ1-3GlcNAcβ1-3Galβ1-3GlcNAcβ-sp2	1.29
	378	Galβ1-3GlcNAcα1-3Galβ1-4GlcNAcβ-sp3	2.17
	379	Galβ1-3GlcNAcβ1-3Galβ1-4GlcNAcβ-sp3	2.17
	380	Galβ1-3GlcNAcα1-6Galβ1-4GlcNAcβ-sp2	1.04
	381	Galβ1-3GlcNAcβ1-6Galβ1-4GlcNAcβ-sp2	1.31
	382	Galβ1-3GalNAcβ1-4Galβ1-4Glcβ-sp3	1.00
	383	Galβ1-4GlcNAcβ1-3Galβ1-4Glcβ-sp2	1.51
	385	Galβ1-4GlcNAcβ1-3Galβ1-4GlcNAcβ-sp3	1.77
	387	Galβ1-4GlcNAcβ1-6Galβ1-4GlcNAcβ-sp2	2.17
	388	Galβ1-3(Galβ1-4GlcNAcβ1-6)GalNAcα-sp3	1.28
	401	Galβ1-3GlcNAcβ1-3Galβ1-3GlcNAcβ-sp3	1.32
	419	3-O-SuGalβ1-4GlcNAcβ1-3Galβ1-4GlcNAcβ-sp3	1.64
	420	4-O-SuGalβ1-4GlcNAcβ1-3Galβ1-4GlcNAcβ-sp3	1.40
	481	Galα1-3Galβ1-4GlcNAcβ1-3Galβ1-4Glcβ-sp4	2.80

	488	Galβ1-4GlcNAcβ1-3(Galβ1-4GlcNAcβ1-6)GalNAcα-sp3	
	489	Galβ1-4GlcNAcβ1-3(GlcNAcβ1-6)Galβ1-4GlcNAc-sp2	1.93
	490	Galβ1-4GlcNAcβ1-6(GlcNAcβ1-3)Galβ1-4GlcNAcβ-sp2	2.54
	498	(Galβ1-4GlcNAcβ1-3)$_3$-sp3	2.10
	499	Galβ1-4GlcNAcβ1-3(Galβ1-4GlcNAcβ1-6)Galβ1-4GlcNAc-sp2	
	501	Galβ1-3GalNAcβ1-3Galα1-4Galβ1-4Glcβ-sp4	
	1A	Galβ1-3GlcNAc	1.74
	1B	Galβ1-4GlcNAc	
	1C	Galβ1-4Gal	
	1D	Galβ1-6GlcNAc	2.06
	1E	Galβ1-3GalNAc	3.07
	1F	Galβ1-3GalNAcβ1-4Galβ1-4Glc	1.00
	1G	Galβ1-3GlcNAcβ1-3Galβ1-4Glc	1.91
	1H	Galβ1-4GlcNAcβ1-3Galβ1-4Glc	2.85
	1I	Galβ1-4GlcNAcβ1-6(Galβ1-4GlcNAcβ1-3)Galβ1-4Glc	6.64
	1J	Galβ1-4GlcNAcβ1-6(Galβ1-3GlcNAcβ1-3)Galβ1-4Glc	3.69
	1K	Galα1-4Galβ1-4Glc	
	1L	GalNAcα1-O-Ser	
	1M	Galβ1-3GalNAcα1-O-Ser	
	1N	Galα1-3Gal	1.28
	1O	Galα1-3Galβ1-4GlcNAc	2.24
	1P	Galα1-3Galβ1-4Glc	4.27
	2A	Galα1-3Galβ1-4Galα1-3Gal	5.18
	2B	Galβ1-6Gal	2.01
	2C	GalNAcβ1-3Gal	3.46
	2D	GalNAcβ1-4Gal	3.09
	2E	Galα1-4Galβ1-4GlcNAc	2.12
	2F	GalNAcα1-3Galβ1-4Glc	1.90
	2G	Galβ1-3GlcNAcβ1-3Galβ1-4GlcNAcβ1-6(Galβ1-3GlcNAcβ1-3)Galβ1-4Glc	2.84
Terminal GlcNAc	10	GlcNAcβ-sp3	1.33
	22	GlcNAcβ-sp4	1.01
	43	6-O-Su-GlcNAcβ-sp3	
	55	3-O-Su-GlcNAcβ-sp3	
	113	GlcNAcβ1-3GalNAcα-sp3	1.03
	114	GlcNAcβ1-3Manβ-sp4	
	115	GlcNAcβ1-4GlcNAcβ-Asn	
	117	GlcNAcβ1-4GlcNAcβ-sp4	
	118	GlcNAcβ1-6GalNAcα-sp3	
	149	GlcNAcβ1-4(6-O-Su)GlcNAcβ-sp2	2.67
	167	GlcNAcβ1-4-[HOOC(CH_3)CH]-3-O-GlcNAcβ-sp4	1.63
	168	GlcNAcβ1--[HOOC(CH_3)CH]-3-O-GlcNAcβ-L-alanyl-D-i-glutaminyl-L-lysine	
	246	GlcNAcβ1-2Galβ1-3GalNAcα-sp3	
	247	GlcNAcβ1-3Galβ1-3GalNAcα-sp3	2.18

	248	GlcNAcβ1-3Galβ1-4Glcβ-sp2	
	250	GlcNAcβ1-3Galβ1-4GlcNAcβ-sp3	1.05
	251	GlcNAcβ1-4Galβ1-4GlcNAcβ-sp2	1.33
	252	GlcNAcβ1-4GlcNAcβ1-4GlcNAcβ-sp4	1.77
	253	GlcNAcβ1-6Galβ1-4GlcNAcβ-sp2	
	255	GlcNAcβ1-3(GlcNAcβ1-6)GalNAcα-sp3	2.10
	395	GlcNAcβ1-3(GlcNAcβ1-6)Galβ1-4GlcNAcβ-sp3	1.54
	493	(GlcNAcβ1-4)$_5$β-sp4	3.07
	503	(GlcNAcβ1-4)$_6$β-sp4	2.76
	504	(A-GN-M)$_2$-3,6-M-GN-GNβ-sp4	1.65
	505	(GN-M)$_2$-3,6-M-GN-GNβ-sp4	1.15
	4A	GlcNAcβ1-4GlcNAc	3.66
	4B	GlcNAcβ1-4GlcNAcβ1-4GlcNAc	3.58
	4C	GlcNAcβ1-4GlcNAcβ1-4GlcNAcβ1-4GlcNAc	3.23
	4D	GlcNAcβ1-4GlcNAcβ1-4GlcNAcβ1-4GlcNAcβ1-4GlcNAcβ1-4GlcNAc	
	4E	GlcNAcβ1-4MurNAc	4.22
Mannosyl	16	Manα-sp3	
	18	Manβ-sp4	
	19	ManNAcβ-sp4	
	47	6-H$_2$PO$_3$Manα-sp3	2.54
	119	Manα1-2Manβ-sp4	2.23
	120	Manα1-3Manβ-sp4	2.73
	121	Manα1-4Manβ-sp4	1.13
	122	Manα1-6Manβ-sp4	2.60
	123	Manβ1-4GlcNAcβ-sp4	1.99
	124	Manα1-2Manα-sp4	3.58
	258	Manα1-3(Manα1-6)Manβ-sp4	1.92
	495	Manα1-6(Manα1-3)Manα1-6(Manα1-3) Manβ-sp4	3.30
	5A	GlcNAcβ1-2Man	1.12
	5B	GlcNAcβ1-2Manα1-6(GlcNAcβ1-2Manα1-3)Man	2.27
	5C	Manα1-2Man	1.14
	5D	Manα1-3Man	2.28
	5E	Manα1-4Man	3.06
	5F	Manα1-6Man	3.30
	5G	Manα1-6(Manα1-3)Man	2.88
	5H	Manα1-6(Manα1-3)Manα1-6(Manα1-3) Man	1.49
Fucosyl	1	Fucα-sp3	1.50
	71	Fucα1-2Galβ-sp3	1.67
	72	Fucα1-3GlcNAcβ-sp3	
	73	Fucα1-4GlcNAcβ-sp3	1.00
	215	Fucα1-2Galβ1-3GlcNAcβ-sp3	
	216	Fucα1-2Galβ1-4GlcNAcβ-sp3	2.80
	217	Fucα1-2Galβ1-3GalNAcα-sp3	

	219	Fucα1-2Galβ1-4Glcβ-sp4	3.66
	226	Fucα1-2(Galα1-3)Galβ-sp3	1.00
	233	Galβ1-3(Fucα1-4)GlcNAcβ-sp3	
	234	Fucα1-3(Galβ1-4)GlcNAcβ-sp3	1.35
	235	Fucα1-2(GalNAcα1-3)Galβ-sp3	1.77
	287	3-O-Su-Galβ1-3(Fucα1-4)GlcNAcβ-sp3	
	288	Fucα1-3(3-O-Su-Galβ1-4)GlcNAcβ-sp3	2.14
	359	Fucα1-2(Galα1-3)Galβ1-3GlcNAcβ-sp3	
	360	Fucα1-2(Galα1-3)Galβ1-4GlcNAcβ-sp3	1.11
	362	Fucα1-2(Galα1-3)Galβ1-3GalNAcα-sp3	1.33
	363	Fucα1-2(Galα1-3)Galβ1-3GalNAcβ-sp3	
	364	Fucα1-3(Galα1-3Galβ1-4)GlcNAcβ-sp3	1.15
	366	Fucα1-2(GalNAcα1-3)Galβ1-3GlcNAcβ-sp3	
	368	Fucα1-2(GalNAcα1-3)Galβ1-4GlcNAcβ-sp3	
	371	Fucα1-2Galβ1-3(Fucα1-4)GlcNAcβ-sp3	1.38
	372	Fucα1-3(Fucα1-2Galβ1-4)GlcNAcβ-sp3	
	392	Fucα1-2(GalNAcα1-6)GalNAcα1-6-sp3	2.84
	479	Fucα1-2Galβ1-3GlcNAcβ1-3Galβ1-4Glcβ-sp4	3.60
	480	Fucα1-2Galβ1-3GlcNAcβ1-3Galβ1-4GlcNAcβ-sp2	3.50
	483	Galα1-3(Fucα1-2)Galβ1-4 (Fucα1-3)GlcNAcβ-sp3	2.27
	496	Fucα1-2Galβ1-3(Fucα1-4)GlcNAcβ1-3Galβ1-4Glcβ-sp4	3.27
	497	Fucα1-3(Fucα1-2Galβ1-4)GlcNAcβ1-3Galβ1-4Glcβ-sp4	3.27
	538	Le^{x}1-6'(Le^{c}1-3')Lac-sp4	1.79
	539	LacNAc1-6'(Le^{d}1-3')Lac-sp4	
	540	Le^{x}1-6'(6'SLN1-3')Lac-sp4	2.59
	541	Le^{x}1-6'(Le^{d}1-3')Lac-sp4	
	542	Le^{c}Le^{x}1-6'(Le^{c}1-3')Lac-sp4	
	543	Le^{x}1-6'(Le^{b}1-3')Lac-sp4	1.90
	7A	Fucα1-2Galβ1-3GlcNAcβ1-3Galβ1-4Glc	1.63
	7B	Galβ1-3(Fucα1-4)GlcNAcβ1-3Galβ1-4Glc	2.03
	7C	Galβ1-4(Fucα1-3)GlcNAcβ1-3Galβ1-4Glc	1.57
	7D	Fucα1-2Galβ1-3(Fucα1-4)GlcNAcβ1-3Galβ1-4Glc	2.56
	7E	Galβ1-3(Fucα1-4)GlcNAcβ1-3Galβ1-4(Fucα1-3)Glc	3.88
	7F	Fucα1-2Gal	
	7G	Fucα1-2Galβ1-4Glc	3.64
	7H	Galβ1-4(Fucα1-3)Glc	2.46
	7I	Galβ1-4(Fucα1-3)GlcNAc	2.43
	7J	Galβ1-3(Fucα1-4)GlcNAc	2.01
	7K	GalNAcα1-3(Fucα1-2)Gal	2.47
	7L	Fucα1-2Galβ1-4(Fucα1-3)Glc	
	7M	Galβ1-3(Fucα1-2)Gal	5.02
	7N	Fucα1-2Galβ1-4(Fucα1-3)GlcNAc	2.54
	7O	Fucα1-2Galβ1-3GlcNAc	3.59
	7P	Fucα1-2Galβ1-3(Fucα1-4)GlcNAc	2.20

	8A	SO3-3Galβ1-3(Fucα1-4)GlcNAc	1.25
	8B	SO3-3Galβ1-4(Fucα1-3)GlcNAc	
	8C	Galβ1-3GlcNAcβ1-3Galβ1-4(Fucα1-3)GlcNAcβ1-3Galβ1-4Glc	
	8D	Galβ1-4(Fucα1-3)GlcNAcβ1-6(Galβ1-3GlcNAcβ1-3)Galβ1-4Glc	
	8E	Galβ1-4(Fucα1-3)GlcNAcβ1-6(Fucα1-2Galβ1-3GlcNAcβ1-3)Galβ1-4Glc	4.32
	8F	Galβ1-4(Fucα1-3)GlcNAcβ1-6(Fucα1-2Galβ1-3(Fucα1-4)GlcNAcβ1-3)Galβ1-4Glc	
	8G	Lacto-N-fucopentaose VI (LNFP VI)	
	8H	Lacto-N-neodifucohexaose I (LNnDFH I)	
	8I	Lacto-N-neodifucohexaose II (LNnDFH II)	
	8J	Trifucosyllacto-N-neoteraose I (TFLNnTI)	
	8K	Monofucosyllacto- N-neohexaose I (MFLNnH I)	
	8L	Difucosyllacto-N-neohexaose I (DFLNnH I)	
	8M	Difucosyllacto-N-neohexaose II (DFLNnH II)	
	8N	Monofucosyl(1-3)-iso-lacto-N-octaose (MFiLNO)	
	8O	Trifucosyl(1-2,1-2,1-3)-iso-lacto-N-octaose (TFiLNO (1-2,1-2,1-3))	5.19
Terminal Sialylated	48	Neu5Acα-sp3	1.77
	49	Neu5Acα-sp9	
	52	Neu5Gcα-sp3	
	54	9-NAc-Neu5Acα-sp3	
	169	Neu5Acα2-3Galβ-sp3	2.16
	170	Neu5Acα2-6Galβ-sp3	
	171	Neu5Acα2-3GalNAcα-sp3	
	172	Neu5Acα2-6GalNAcα-sp3	
	174	Neu5Gcα2-6GalNAcα-sp3	3.46
	186	Neu5Acα2-8Neu5Acα2-sp3	
	205	Neu5Acα2-6GalNAcβ-sp3	
	206	Neu5Gcα2-3Gal-sp3	3.71
	289	Galα1-3(Neu5Acα2-6)GalNAcα-sp3	1.42
	290	Galβ1-3(Neu5Acα2-6)GalNAcα-sp3	
	292	Neu5Acα2-3Galβ1-3GalNAcα-sp3	1.70
	293	Neu5Acα2-3Galβ1-4Glcβ-sp3	
	294	Neu5Acα2-3Galβ1-4Glcβ-sp4	2.18
	295	Neu5Acα2-6Galβ1-4Glcβ-sp2	
	298	Neu5Acα2-3Galβ1-4GlcNAcβ-sp3	1.22
	299	Neu5Acα2-3Galβ1-3GlcNAcβ-sp3	1.58
	300	Neu5Acα2-6Galβ1-4GlcNAcβ-sp3	1.55
	303	Neu5Gcα2-3Galβ1-4GlcNAcβ-sp3	
	304	Neu5Gcα2-6Galβ1-4GlcNAcβ-sp3	1.42
	306	9-NAc-Neu5Acα2-6Galβ1-4GlcNAcβ-sp3	
	315	Neu5Acα2-3Galβ1-4-(6-O-Su)GlcNAcβ-sp3	1.77
	317	Neu5Acα2-3Galβ1-3-(6-O-Su)GalNAcβ-sp3	
	318	Neu5Acα2-6Galβ1-4-(6-O-Su)GlcNAcβ-sp3	1.62
	319	Neu5Acα2-3-(6-O-Su)Galβ1-4GlcNAcβ-sp3	1.00

	321	(Neu5Acα2-8)$_3$-sp3	2.55
	323	Neu5Acα2-6Galβ1-3GlcNAc-sp3	
	324	Neu5Acα2-6Galβ1-3(6-O-Su)GlcNAc-sp3	
	331	Neu5Gcα2-3Galβ1-3GlcNAcβ-sp3	
	421	Neu5Acα2-3(GalNAcβ1-4)Galβ1-4Glcβ-sp2	2.38
	422	Neu5Acα2-3Galβ1-4GlcNAcβ1-3Galβ-sp3	1.86
	423	Fucα1-3(Neu5Acα2-3Galβ1-4)GlcNAcβ-sp3	
	426	Neu5Acα2-3Galβ1-3(Fucα1-4)GlcNAcβ-sp3	2.18
	428	Fucα1-3(Neu5Acα2-3Galβ1-4)6-O-Su-GlcNAcβ-sp3	1.96
	429	Fucα1-3(Neu5Acα2-3(6-O-Su)Galβ1-4)GlcNAcβ-sp3	2.12
	433	Neu5Acα2-3Galβ1-3(Neu5Acα2-6)GalNAcα-sp3	
	434	Neu5Acα2-8Neu5Acα2-3Galβ1-4Glcβ-sp4	2.30
	527	Neu5Acα2-3Galβ1-4GlcNAcβ1-3Galβ1-4GlcNAcβ-sp2	4.28
	528	Fucα1-3(Neu5Acα2-3Galβ1-4)GlcNAcβ1-3Galβ-sp3	3.32
	529	Galβ1-3(Neu5Acα2-6)GlcNAcβ1-3Galβ1-4Glcβ-sp4	1.98
	531	Neu5Acα2-8Neu5Acα2-3(GalNAcβ1-4)Galβ1-4Glc-sp2	1.28
	532	Neu5Acα2-8Neu5Acα2-8Neu5Acα2-3Galβ1-4Glc-sp2	1.85
	533	(Neu5Acα2-8)2Neu5Acα2-3(GalNAcβ1-4)Galβ1-4Glc-sp2	
	534	Neu5Acα2-3Galβ1-4GlcNAcβ1-3Galβ1-4GlcNAcβ-sp3	3.32
	536	Neu5Acα2-3Galβ1-3GlcNAcβ1-3Galβ1-4Glcβ-sp4	
	537	Neu5Acα2-3Galβ1-4GlcNAcβ1-3Galβ1-4Glcβ-sp4	2.46
	627	(Sia2-6A-GN-M)$_2$-3,6-M-GN-GNβ-sp4	2.46
	10A	Neu5Acα2-3Galβ1-3(Fucα1-4)GlcNAc	
	10B	Neu5Acα2-3Galβ1-4(Fucα1-3)GlcNAc	2.80
	10C	Neu5Acα2-3Galβ1-3GlcNAcβ1-3Galβ1-4Glc	
	10D	Galβ1-4(Fucα1-3)GlcNAcβ1-6(Neu5Acα2-6Galβ1-4GlcNAcβ1-3)Galb1-4Glc	
	10E	Disialyl-TF	2.10
	10H	Sialyllacto-N-fucopentaose VI (SLNFPVI)	7.56
	10K	Neu5Acα2-3Galβ1-4GlcNAc	
	10L	Neu5Acα2-6Galβ1-4GlcNAc	5.45
	10M	Neu5Acα2-3Galβ1-3GlcNAcβ1-3Galβ1-4Glc	4.50
	10N	Galβ1-3(Neu5Acα2-6)GlcNAcβ1-3Galβ1-4Glc	4.86
	10O	Neu5Acα2-6Galβ1-4GlcNAcβ1-3Galβ1-4Glc	
	10P	Neu5Acα2-3Galβ1-3(Neu5Acα2-6)GlcNAcβ1-3Galβ1-4Glc	4.01
	11A	Neu5Acα2-3Galβ1-4Glc	2.85
	11B	Neu5Acα2-6Galβ1-4Glc	3.14
	11C	(Neu5Acα2-8Neu5Ac)n (n<50)	4.10
	11D	Neu5Acα2-6Galβ1-4GlcNAcβ1-2Manα1-6(Neu5Acα2-6Galβ1-4GlcNAcβ1-2Manα1-6)Manβ1-4GlcNAcβ1-4GlcNAc-Asn	8.73
GalNAc	4	GalNAcα-sp0	
	5	GalNAcα-sp3	
	6	GalNAcβ-sp3	1.75
	38	3-O-Su-GalNAcα-sp3	
	101	GalNAcα1-3GalNAcβ-sp3	

	102	GalNAcα1-3Galβ-sp3	1.29
	103	GalNAcα1-3GalNAcα-sp3	
	104	GalNAcβ1-3Galβ-sp3	
	106	GalNAcβ1-4GlcNAcβ-sp3	
	192	GalNAcβ1-4(6-O-Su)GlcNAcβ-sp3	
	193	3-O-Su-GalNAcβ1-4GlcNAcβ-sp3	2.38
	194	6-O-Su-GalNAcβ1-4GlcNAcβ-sp3	1.00
	195	6-O-Su-GalNAcβ1-4-(3-O-Su)GlcNAcβ-sp3	2.87
	196	3-O-Su-GalNAcβ1-4(3-O-Su)-GlcNAcβ-sp3	
	197	3,6-O-Su_2-GalNAcβ1-4GlcNAcβ-sp3	3.86
	198	4,6-O-Su_2-GalNAcβ1-4GlcNAcβ-sp3	6.43
	199	4,6-O-Su_2-GalNAcβ1-4-(3-O-Ac)GlcNAcβ-sp3	
	200	4-O-Su-GalNAcβ1-4GlcNAcβ-sp3	2.18
	202	6-O-Su-GalNAcβ1-4(6-O-Su)GlcNAcβ-sp3	
	204	4-O-Su-GalNAcβ1-4GlcNAcβ-sp2	
	238	GalNAcβ1-4Galβ1-4Glcβ-sp3	
	389	GalNacβ1-3GalαGalβ1-4Glcβ-sp3	1.58
Terminal Glucose	7	Glcα-sp3	1.18
	9	Glcβ-sp3	1.43
	46	6-H_2PO_3Glcβ-sp4	
	110	Glcα1-4Glcβ-sp3	
	111	Glcβ1-4Glcβ-sp4	1.24
	112	Glcβ1-6Glcβ-sp4	
	240	$(Glc\alpha1\text{-}4)_3\beta$-sp4	1.26
	241	$(Glc\alpha1\text{-}6)_3\beta$-sp4	1.45
	390	$(Glc\alpha1\text{-}4)_4\beta$-sp4	2.29
	391	$(Glc\alpha1\text{-}6)_4\beta$-sp4	2.78
	492	$(Glc\alpha1\text{-}6)_5\beta$-sp4	2.36
	502	$(Glc\alpha1\text{-}6)_6\beta$-sp4	
Glycosaminoglycan digests (low molecular weight)	12A	Neocarratetraose-41, 3-di-*O*-sulphate (Na^+)	5.90
	12B	Neocarratetraose-41-*O*-sulphate (Na^+)	4.12
	12C	Neocarrahexaose-24,41, 3, 5-tetra-*O*-sulphate (Na^+)	5.07
	12D	Neocarrahexaose-41, 3, 5-tri-*O*-sulphate (Na^+)	3.96
	12E	Neocarraoctaose-41, 3, 5, 7-tetra-*O*-sulphate (Na^+)	6.38
	12F	Neocarradecaose-41, 3, 5, 7, 9-penta-*O*-sulphate (Na^+)	1.88
	12G	ΔUA-2S → GlcNS-6S Na_4 (I-S)	4.78
	12H	ΔUA → GlucNS-6S Na_3 (II-S)	2.36
	12I	ΔUA → 2S-GlcNS Na_3 (III-S)	3.38
	12J	ΔUA → 2S-GlcNAc-6S Na_3 (I-A)	3.59
	12K	ΔUA → GlcNAc-6S Na_2 (II-A)	4.30
	12L	ΔUA → 2S-GlcNAc Na_2 (III-A)	1.52

	12M	ΔUA → GlcNAc Na (IV-A)	3.22
	12N	ΔUA → GalNAc-4S Na_2 (Δ Di-4S)	
	12O	ΔUA → GalNAc-6S Na2 (Δ Di-6S)	6.27
	12P	ΔUA → GalNAc-4S,6S Na_3 (Δ Di-disE)	4.67
	13A	ΔUA → 2S-GalNAc-4S Na_2 (Δ Di-disB)	3.13
	13B	ΔUA → 2S-GalNAc-6S Na_3 (Δ Di-disD)	
	13C	ΔUA → 2S-GalNAc-4S-6S Na_4 (Δ Di-tisS)	
	13D	ΔUA → 2S-GalNAc-6S Na_2 (ΔDi-UA2S)	4.78
	13E	ΔUA → GlcNAc Na (Δ Di-HA)	3.17
Glycosaminoglycans (high molecular weight)	13F	(GlcAβ1-3GlcNAcβ1-4)n (n=4)	3.08
	13G	(GlcAβ1-3GlcNAcβ1-4)n (n=8)	2.46
	13H	(GlcAβ1-3GlcNAcβ1-4)n (n=10)	3.61
	13I	(GlcAβ1-3GlcNAcβ1-4)n (n=12)	
	13J	(GlcA/IdoAα/β1-4GlcNAcα1-4)n (n=200)	3.93
	13K	(GlcA/IdoAβ1-3(±4/6S)GalNAcβ1-4)n (n<250)	4.61
	13L	((±2S)GlcA/IdoAα/β1-3(±4S)GalNAcβ1-4)n (n<250)	4.16
	13M	(GlcA/IdoAβ1-3(±6S)GalNAcβ1-4)n (n<250)	1.54
	13N	(GlcAβ1-3GlcNAcβ1-4)n (n=4)	5.38
	13O	(GlcAβ1-3GlcNAcβ1-4)n (n=6)	2.99
	13P	(GlcAβ1-3GlcNAcβ1-4)n (n=8)	2.65
	14A	(GlcAβ1-3GlcNAcβ1-4)n (n=10)	
	14B	(GlcAβ1-3GlcNAcβ1-4)n (n=12)	2.18
	14C	(GlcAβ1-3GlcNAcβ1-4)n (n=14)	
	14D	(GlcAβ1-3GlcNAcβ1-4)n (n=16)	3.42
	14E	HA-30,000 Da	4.20
	14F	HA-107,000 Da	5.34
	14G	HA-190,000 Da	3.75
	14H	HA-220,000 Da	3.00
	14I	HA-1,600,000 Da	1.25
	14J	Heparan Sulfate	1.69
	14K	(Glcβ1-3Glcβ1-3)n	1.02
Other	14	GlcN(Gc)β-sp4	
	15	$HOCH_2(HOCH)_4CH_2NH_2$	
	20	Rhaα-sp3	1.60
	44	GlcAα-sp3	
	45	GlcAβ-sp3	1.10
	164	GlcAβ1-3GlcNAcβ-sp3	2.55
	165	GlcAβ1-3Galβ-sp3	1.85
	166	GlcAβ1-6Galβ-sp3	5.10
	625	(GlcAβ1-4GlcNAcβ1-3)$_8$-NH_2-ol	2.93
