# Supplementary material for: Glycointeractome of Neisseria gonorrhoeae: Identification of Host Glycans Targeted by the Gonococcus To Facilitate Adherence to Cervical and Urethral Epithelial Cells
Source: mBio. 2019 Jul 9;10(4):e01339-19. doi: 10.1128/mBio.01339-19 (PMC6747729; doi:10.1128/mBio.01339-19)
Supplement: FIG S1 [file mBio.01339-19-sf001.pdf]

The glycointeractome of *Neisseria gonorrhoeae* – identification of host glycans targeted by the gonococcus to facilitate adherence to cervical and urethral epithelial cells

Evgeny A. Semchenko, Arun V. Everest-Dass, Freda E.-C. Jen, Tsitsi D. Mubaiwa, Christopher J. Day and Kate L. Seib

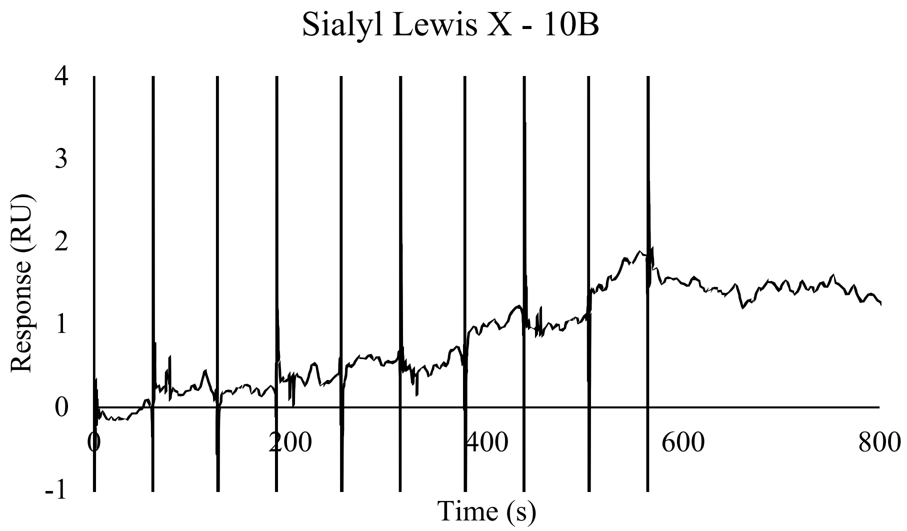

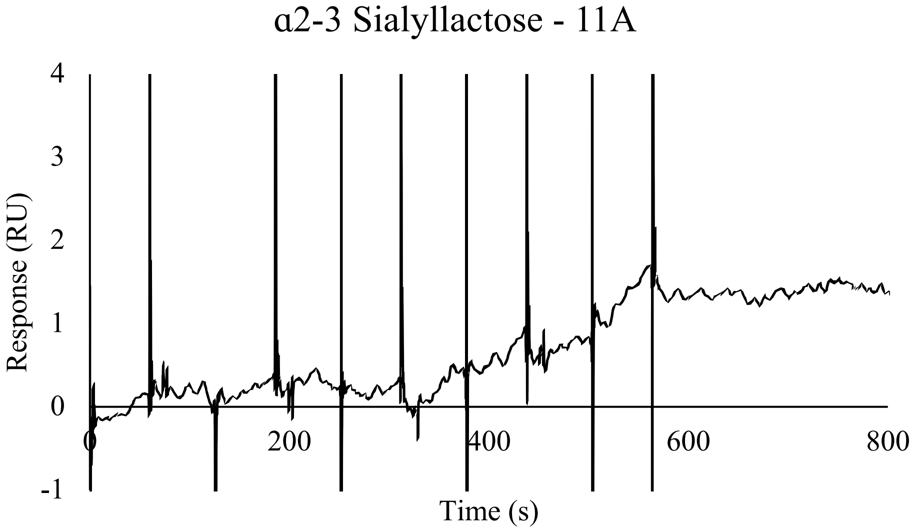

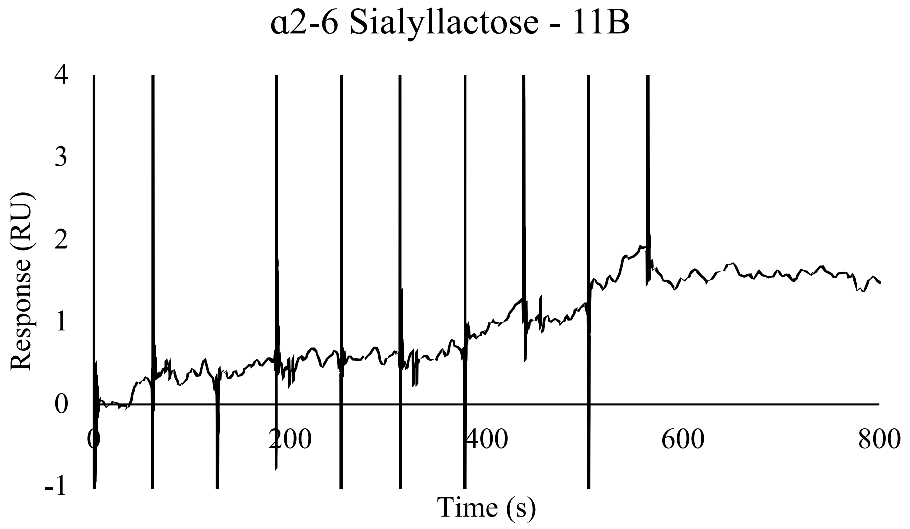
α2-6 Sialyllactose - 11B
Response (RU)
4
3
2
1
0
-1
0
200
400
600
800
Time (s)

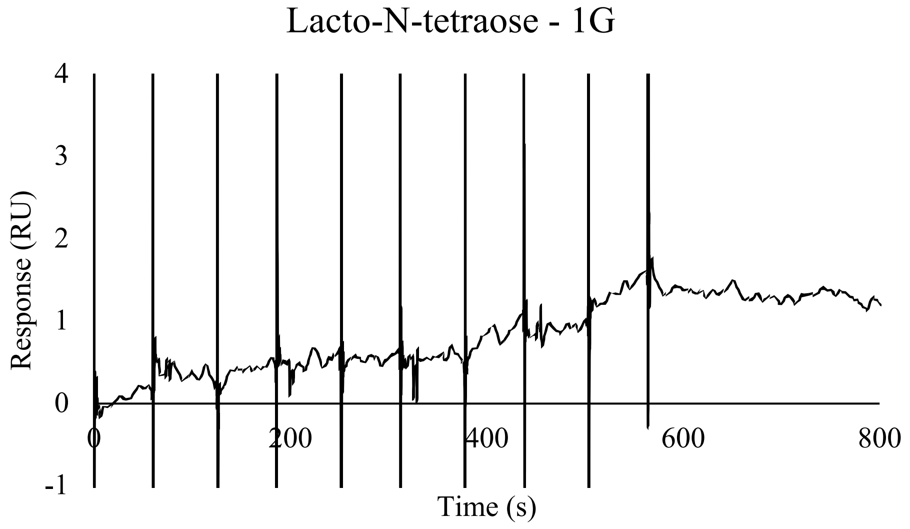
Lacto-N-tetraose - 1G
Response (RU)
4
3
2
1
0
-1
0
200
400
600
800
Time (s)

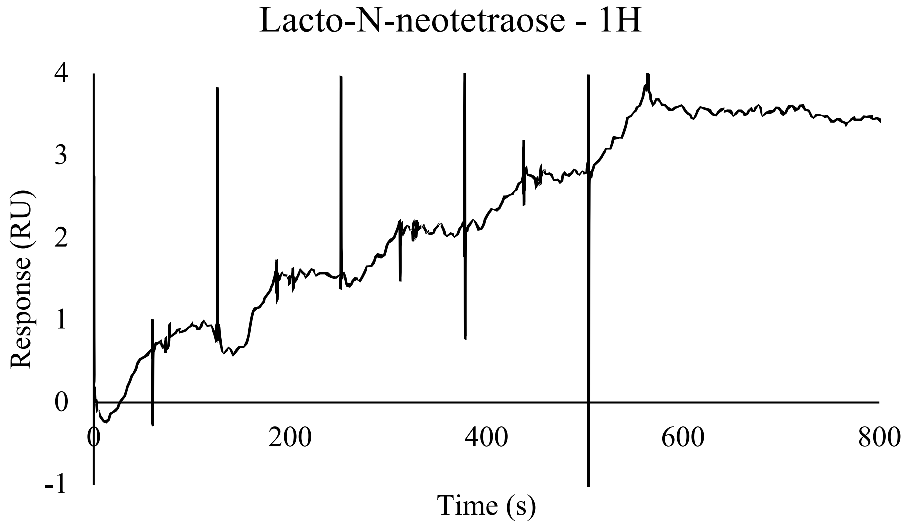
Lacto-N-neotetraose - 1H
Response (RU)
4
3
2
1
0
-1
0
200
400
600
800
Time (s)

Hexaacetyl chitohexaose - 4D

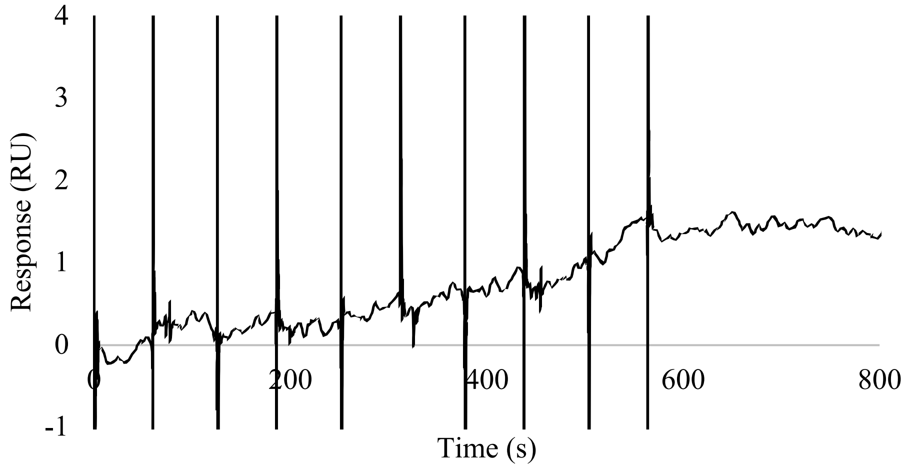

α1-2 Mannobiose - 5C

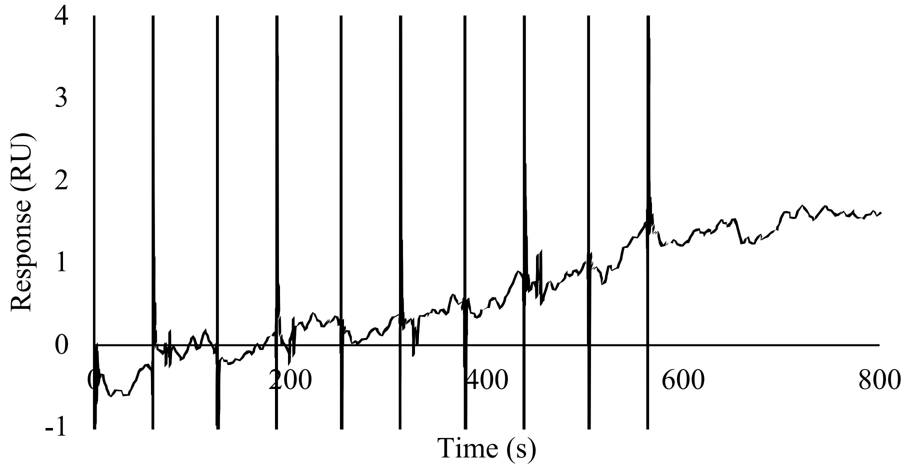

α1-3 Mannobiose - 5D

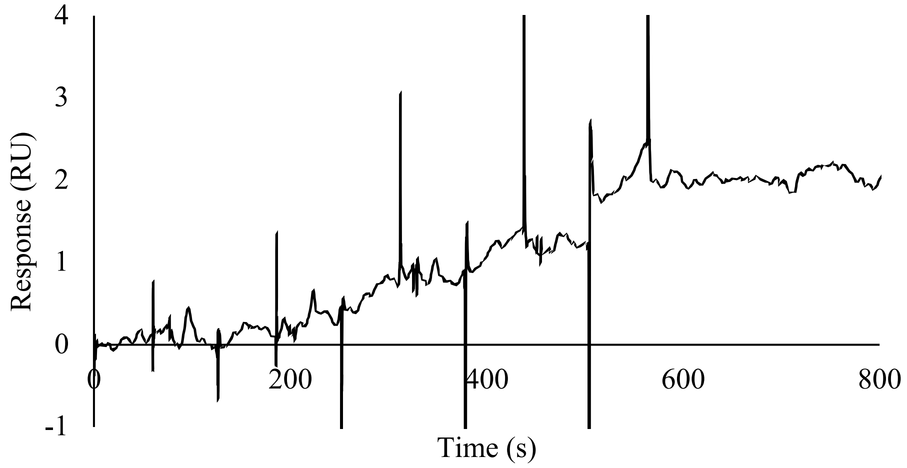

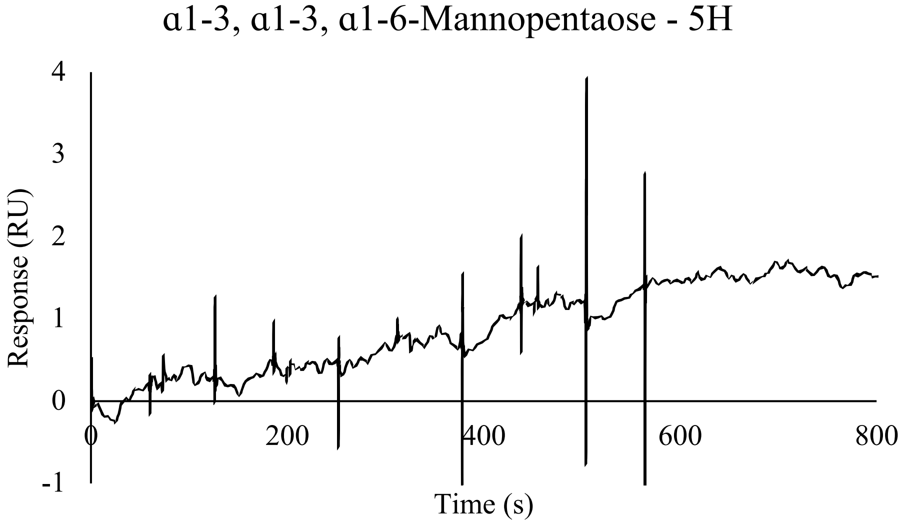
ɑ1-3, ɑ1-3, ɑ1-6-Mannopentaose - 5H
Response (RU)
4
3
2
1
0
-1
0
200
400
600
800
Time (s)

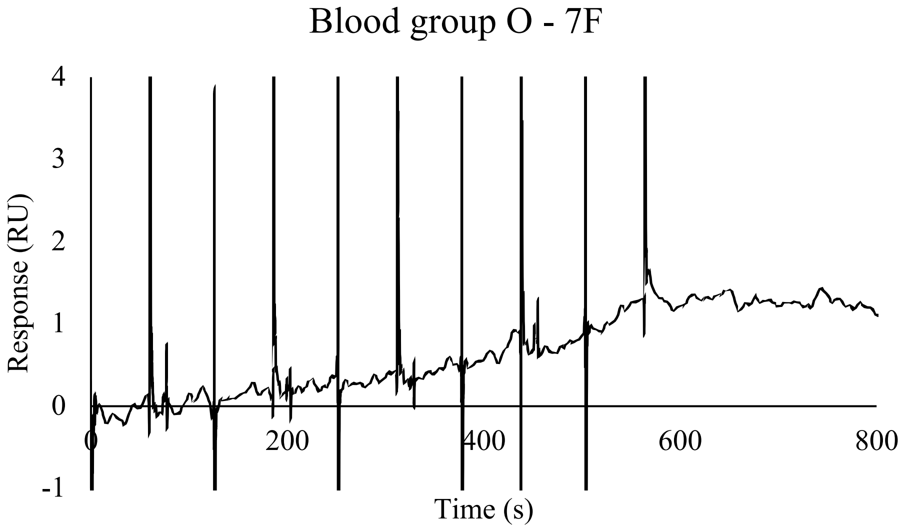
Blood group O - 7F
Response (RU)
4
3
2
1
0
-1
0
200
400
600
800
Time (s)

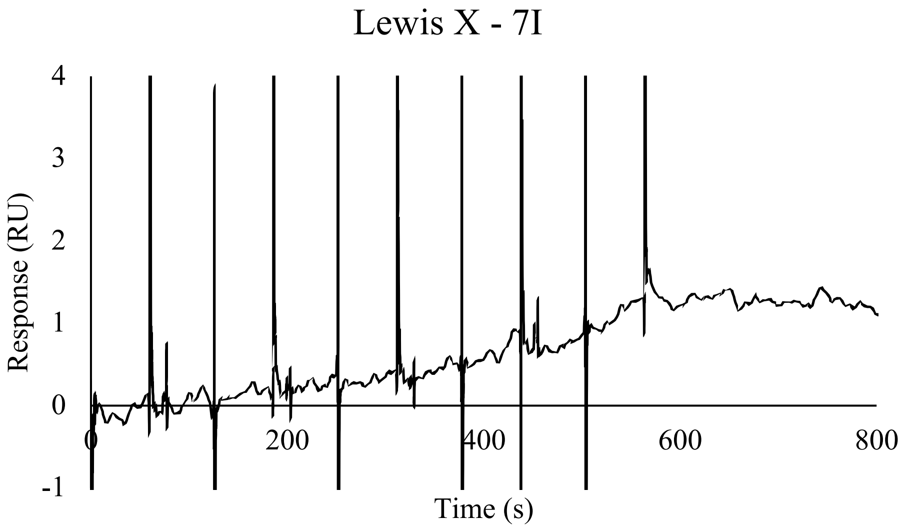
Lewis X - 7I
Response (RU)
4
3
2
1
0
-1
0
200
400
600
800
Time (s)

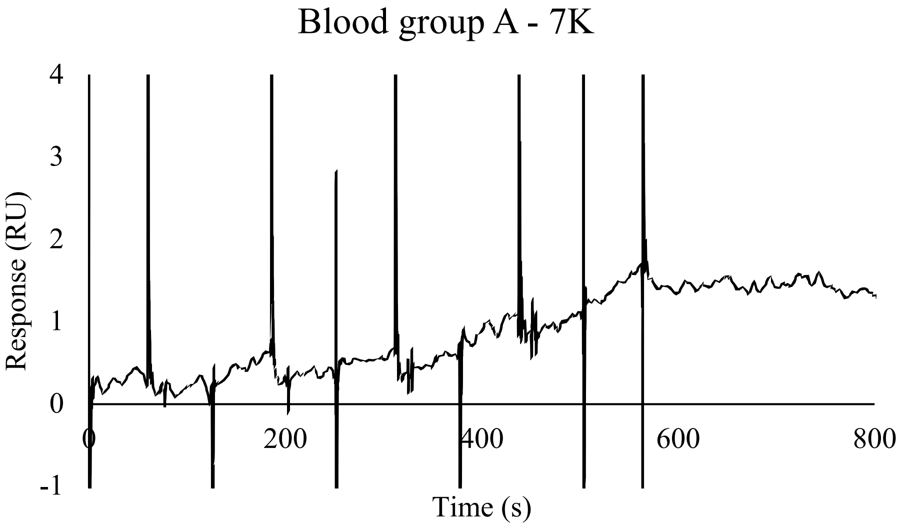
Blood group A - 7K
Response (RU)
4
3
2
1
0
-1
0
200
400
600
800
Time (s)

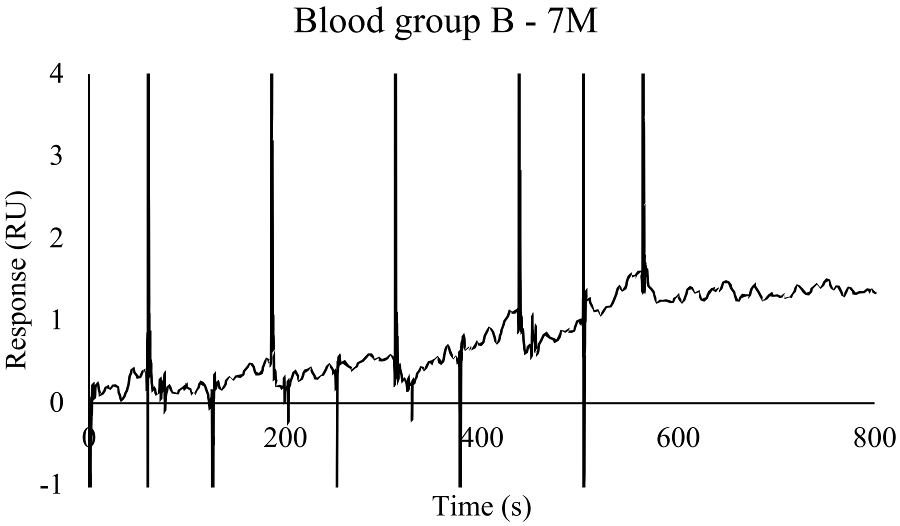
Blood group B - 7M
Response (RU)
4
3
2
1
0
-1
0
200
400
600
800
Time (s)

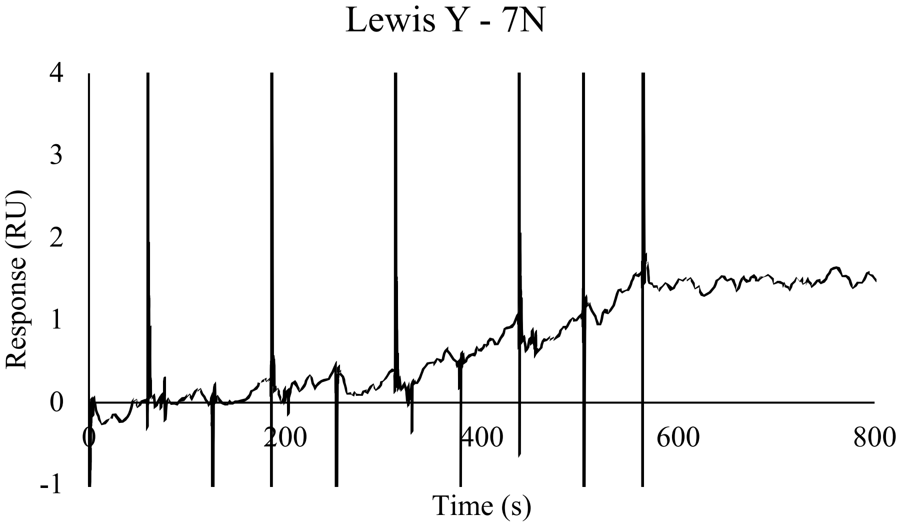
Lewis Y - 7N
Response (RU)
4
3
2
1
0
-1
0
200
400
600
800
Time (s)

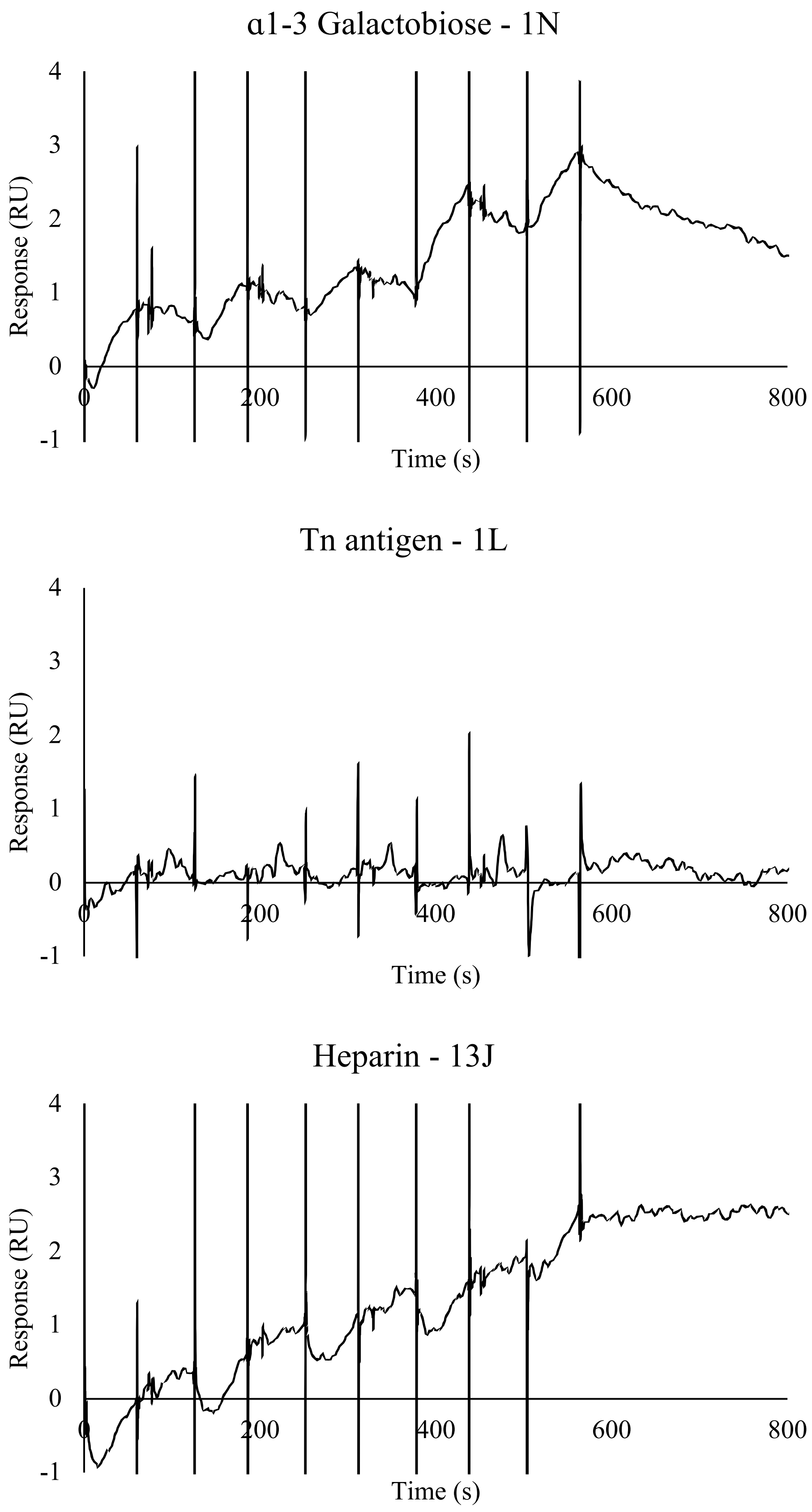

Figure S1. Glycan binding by *N. gonorrhoeae.*
Representative sensorgrams from surface plasmon resonance (SPR) analysis of whole-cell *N. gonorrhoeae* and selected glycans.
