# Supplementary material for: Glycointeractome of Neisseria gonorrhoeae: Identification of Host Glycans Targeted by the Gonococcus To Facilitate Adherence to Cervical and Urethral Epithelial Cells
Source: mBio. 2019 Jul 9;10(4):e01339-19. doi: 10.1128/mBio.01339-19 (PMC6747729; doi:10.1128/mBio.01339-19)
Supplement: FIG S2 [file mBio.01339-19-sf002.pdf]

The glycointeractome of *Neisseria gonorrhoeae* – identification of host glycans targeted by the gonococcus to facilitate adherence to cervical and urethral epithelial cells

Evgeny A. Semchenko, Arun V. Everest-Dass, Freda E.-C. Jen, Tsitsi D. Mubaiwa, Christopher J. Day and Kate L. Seib

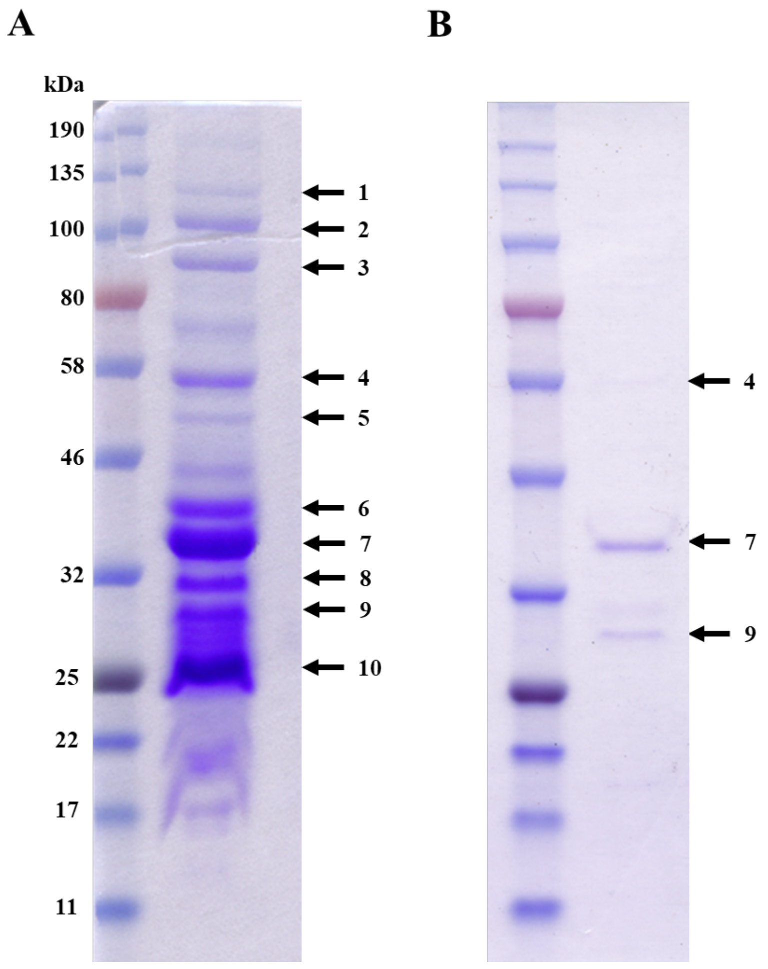

Figure S2. Gonococcal mannose binding proteins.
(A) *N. gonorrhoeae* 1291 mannose-binding proteins eluted from D-mannose-coupled agarose and resolved on 4-12% Bis-Tris SDS-PAGE. (B) The negative control (cell membrane proteins with D-mannose added as a binding competitor prior to incubation with D-mannose agarose) contained negligible protein, suggesting that most of the eluted proteins interact with mannose in specific manner. Bands (1-10), were excised from the gel for protein identification using mass spectrometry.
