# Supplementary material for: Glycointeractome of Neisseria gonorrhoeae: Identification of Host Glycans Targeted by the Gonococcus To Facilitate Adherence to Cervical and Urethral Epithelial Cells
Source: mBio. 2019 Jul 9;10(4):e01339-19. doi: 10.1128/mBio.01339-19 (PMC6747729; doi:10.1128/mBio.01339-19)
Supplement: FIG S3 [file mBio.01339-19-sf003.pdf]

The glycointeractome of *Neisseria gonorrhoeae* – identification of host glycans targeted by the gonococcus to facilitate adherence to cervical and urethral epithelial cells

Evgeny A. Semchenko, Arun V. Everest-Dass, Freda E.-C. Jen, Tsitsi D. Mubaiwa, Christopher J. Day and Kate L. Seib

A

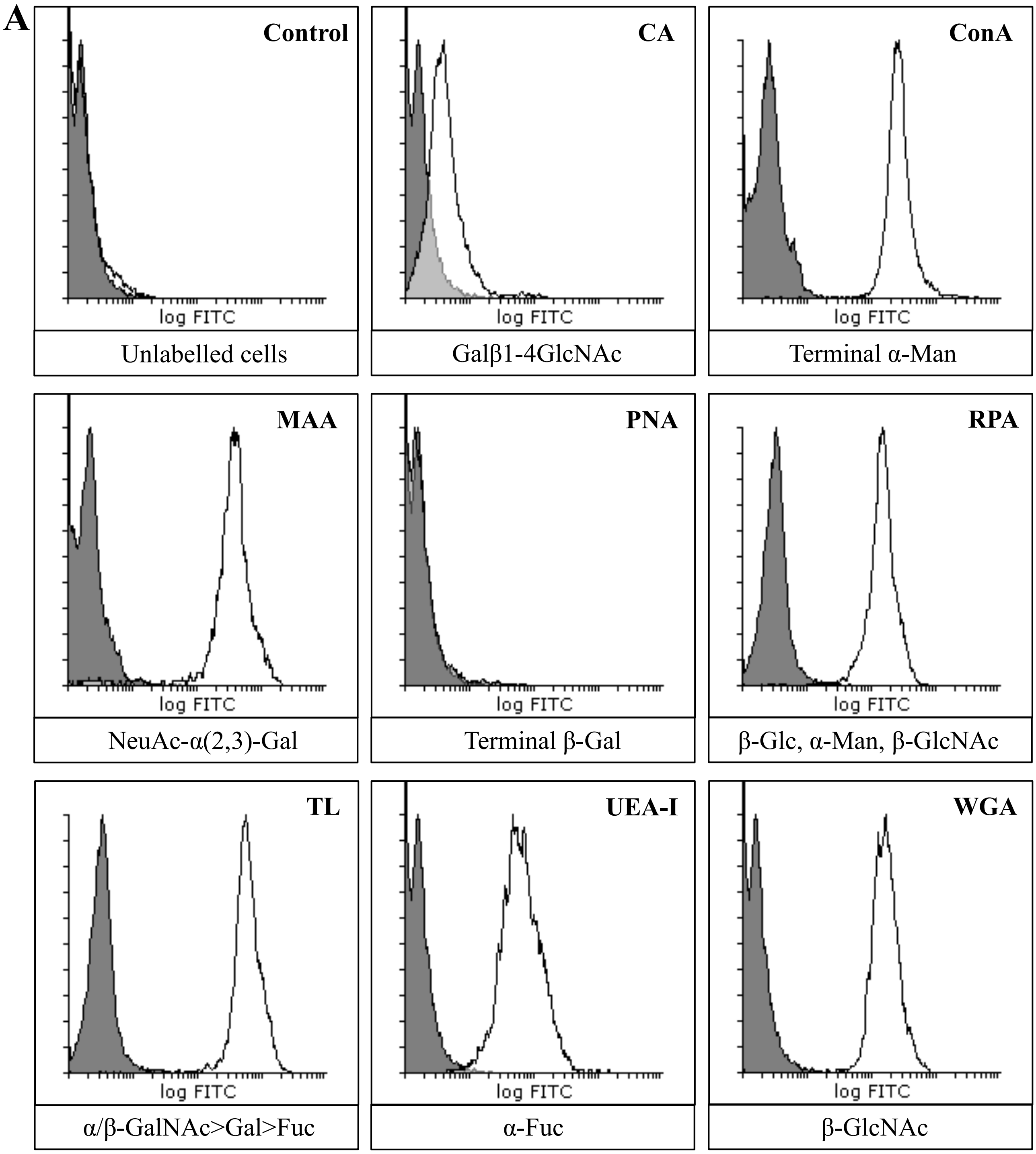

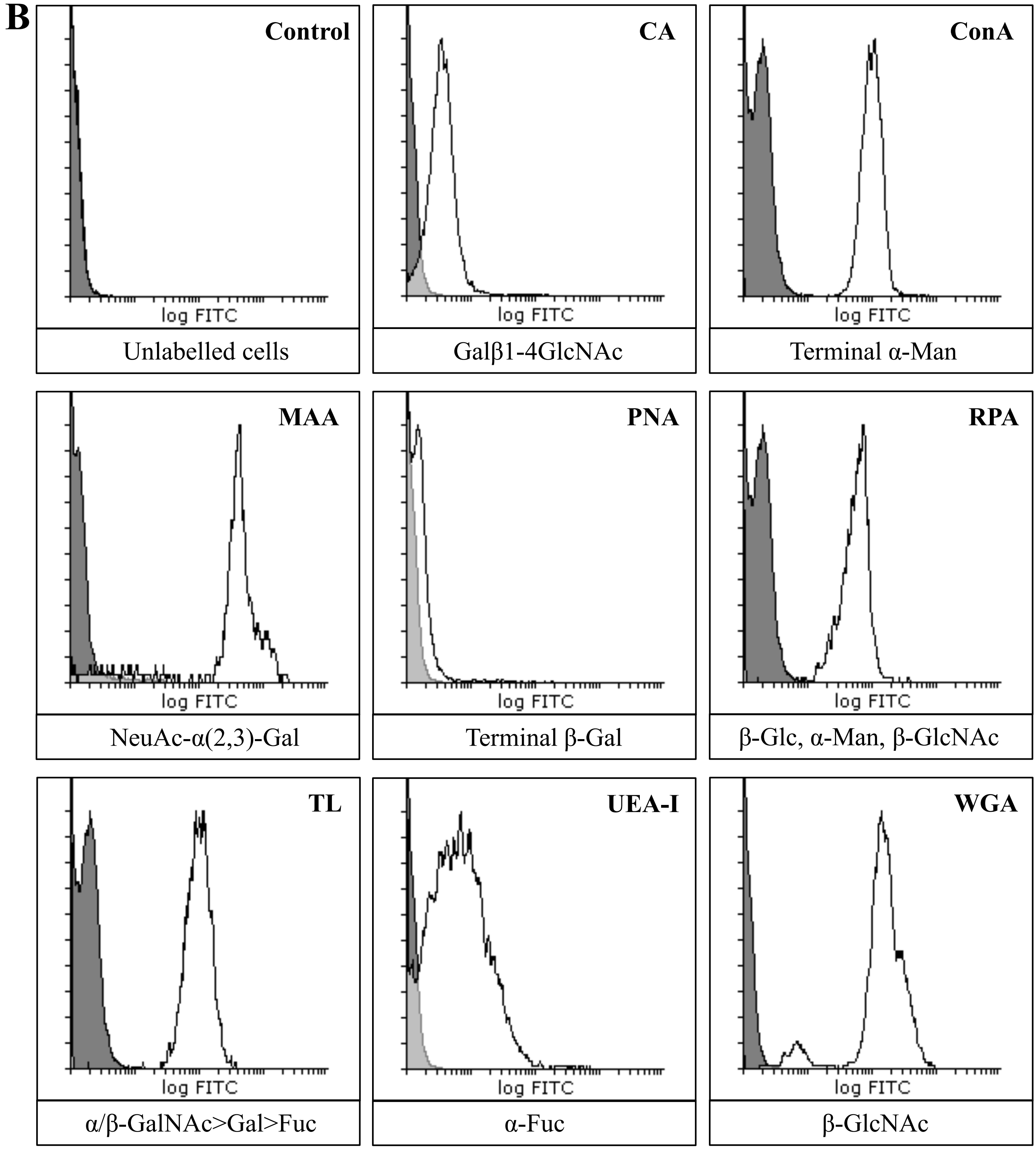

Figure S3. Analysis of lectin binding by epithelial cells.
Identification of **(A)** urethral epithelial cell (tUEC) and **(B)** cervical epithelial cell (tCX) surface glycans with fluorescein isothiocyanate (FITC) labelled lectins using flow cytometry. Control histogram shows the overlay of the non-labelled cell population and cells incubated with the final elution of the buffer that followed the clean-up of the FITC labelling of lectins. This demonstrates that no unreacted, residual fluorescein molecules were present in any of the lectin samples. Each panel shows a histogram from an individual experiment with a lectin (top right corner) and the glycan it recognises (bottom label). Lectin abbreviations: CA - *Colchicum autumnale* lectin, ConA – *Canavalia ensiformis* lectin, MAA - *Maackia amurensis* lectin, PNA – *Arachis hypogaea* lectin, RPA - *Robinia pseudoacacia* lectin, TL - *Tulipa* sp. lectin, UEA-I – *Ulex europaeus* lectin, WGA - *Triticum vulgaris* lectin.
